# Safety, Immunogenicity and Efficacy of Prime-Boost Vaccination with ChAd63 and MVA Encoding ME-TRAP against *Plasmodium falciparum* Infection in Adults in Senegal

**DOI:** 10.1371/journal.pone.0167951

**Published:** 2016-12-15

**Authors:** Victorine A. Mensah, Aly Gueye, Magatte Ndiaye, Nick J. Edwards, Danny Wright, Nicholas A. Anagnostou, Massamba Syll, Amy Ndaw, Annie Abiola, Carly Bliss, Jules-François Gomis, Ines Petersen, Caroline Ogwang, Tandakha Dieye, Nicola K. Viebig, Alison M. Lawrie, Rachel Roberts, Alfredo Nicosia, Babacar Faye, Oumar Gaye, Odile Leroy, Egeruan B. Imoukhuede, Katie J. Ewer, Philip Bejon, Adrian V. S. Hill, Badara Cisse, MVVC group

**Affiliations:** 1 Parasitology department, Faculty of Medicine University Cheikh Anta Diop, Dakar, Senegal; 2 Centre for Clinical Vaccinology and Tropical Medicine, The Jenner Institute, University of Oxford, Churchill Hospital, Oxford, United Kingdom; 3 European Vaccine Initiative, Heidelberg, Germany; 4 Kenya Medical Research Institute, Kilifi, Kenya; 5 ReiThera (formerly Okairos), Rome, Italy; 6 CEINGE, via Gaetano Salvatore, Naples, Italy; 7 London School of Hygiene and Tropical Medicine, London United Kingdom; Sanaria,. Inc, UNITED STATES

## Abstract

Malaria transmission is in decline in some parts of Africa, partly due to the scaling up of control measures. If the goal of elimination is to be achieved, additional control measures including an effective and durable vaccine will be required. Studies utilising the prime-boost approach to deliver viral vectors encoding the pre-erythrocytic antigen ME-TRAP (multiple epitope thrombospondin-related adhesion protein) have shown promising safety, immunogenicity and efficacy in sporozoite challenge studies. More recently, a study in Kenyan adults, similar to that reported here, showed substantial efficacy against *P*. *falciparum* infection. One hundred and twenty healthy male volunteers, living in a malaria endemic area of Senegal were randomised to receive either the Chimpanzee adenovirus (ChAd63) ME-TRAP as prime vaccination, followed eight weeks later by modified vaccinia Ankara (MVA) also encoding ME-TRAP as booster, or two doses of anti-rabies vaccine as a comparator. Prior to follow-up, antimalarials were administered to clear parasitaemia and then participants were monitored by PCR for malaria infection for eight weeks. The primary endpoint was time-to-infection with *P*. *falciparum* malaria, determined by two consecutive positive PCR results. Secondary endpoints included adverse event reporting, measures of cellular and humoral immunogenicity and a meta-analysis of combined vaccine efficacy with the parallel study in Kenyan adults.We show that this pre-erythrocytic malaria vaccine is safe and induces significant immunogenicity, with a peak T-cell response at seven days after boosting of 932 Spot Forming Cells (SFC)/10^6^ Peripheral Blood Mononuclear Cells(PBMC) compared to 57 SFC/ 10^6^ PBMCs in the control group. However, a vaccine efficacy was not observed: 12 of 57 ME-TRAP vaccinees became PCR positive during the intensive monitoring period as compared to 13 of the 58 controls (P = 0.80). This trial confirms that vaccine efficacy against malaria infection in adults may be rapidly assessed using this efficient and cost-effective clinical trial design. Further efficacy evaluation of this vectored candidate vaccine approach in other malaria transmission settings and age-de-escalation into the main target age groups for a malaria vaccine is in progress.

## Introduction

Malaria transmission has been on the decline in many parts of Africa in association with scaling up of effective control measures [1–3]. Elimination and subsequent eradication of malaria has been the subject of discourse for several years [4,5]. However, if the goal of eradication is to be achieved, additional control measures including effective and durable vaccines will be required.

The pre-erythocytic malaria vaccine RTS, S targeting the circumsporozoite protein, is the most advanced vaccine in clinical development. In the recently published Phase III clinical trial in over 15,000 infants and children in several African countries, the efficacy of RTS, S, with AS01 as adjuvant, in young children was about 50% during the first year [6–11], but lower at 34–36% over 4 years [12]. Efficacy declines with time and was much lower against severe malaria after 3 years of follow-up in younger infants (10–17%)[11]. This partial efficacy necessitates the development of other candidate vaccines, which could be used alone or in combination with RTS, S. Studies in multiple disease areas show that vectored vaccines induce more potent T cell responses that can provide significant efficacy. Heterologous prime-boost immunisation with chimpanzee adenovirus 63 (ChAd63) and modified vaccinia virus Ankara (MVA) vectored vaccines is a vaccination strategy recently shown to induce cell-mediated responses against several *P*. *falciparum* malaria antigens [13–15]. ChAd63-MVA expressing the *P*. *falciparum* pre-erythrocytic antigen ME-TRAP (multiple epitope string thrombospondin-related adhesion protein) is one of the most advanced malaria vaccine candidates, capable of inducing sterile protection in 21% of malaria naïve adults following controlled human malaria infection (CHMI)[16].

Recently in Sukuta, The Gambia and Kilifi, Kenya, two phase Ib dose-escalation clinical trials were undertaken to assess the safety and immunogenicity of this approach in 46 healthy malaria-exposed adults as previously, immunogenicity had only been described in predominantly Caucasian, malaria-naïve adults. These two studies showed encouraging results, with both vaccines shown to be safe and well tolerated, with high-level T cell responses induced (median > 1300 Spot Forming Cells/million Peripheral Blood Monuclear Cells [14,17]. The T cell responses appeared to be the most potent reported in Africa for any vaccine type and were therefore consistent with the growing evidence that ChAd63 and MVA were promising vectors for clinical use. In Kilifi in Eastern Kenya, malaria is endemic with two transmission seasons (April-June and October-December) while in Senegal the transmission is made of one short and intense transmission season (July to August-November). It was therefore useful to assess the safety, immunogenicity and efficacy of candidate vaccine in semi-immune West African adults exposed to different malaria transmission conditions.

The use of PCR detection has been found to be feasible and cost-effective in studies conducted in The Gambia and more recently in Kenya [18,19]. These clinical efficacy trials measure time to PCR-detected infection after drug clearance of parasites. Efficacy against infection is measured over an 8-week follow-up period starting 14 days after the final vaccination (7 days after drug clearance). In a Phase IIb trial of vaccine efficacy in 120 Kenyan adults, efficacy against infection was 67% (95%CI 33% to 83%), p = 0.002, over 8 weeks of follow-up [19].

Using malaria infection detected by PCR as an endpoint for efficacy, we assessed the safety, immunogenicity and efficacy of priming with the same vaccines and doses, ChAd63 ME-TRAP (5 x 10^5^ vp) vaccine and boosting with MVA ME-TRAP (2 x 10^8^ pfu), 8 weeks apart in healthy adult volunteers in Senegal.

## Methods

**Registration:** Pan African Clinical Trial Registry (Ref: PACTR 201-303-000-499-409); http://www.pactr.org

### Study setting

The study was conducted in the peri-urban area of Dakar in Senegal, West Africa, in one of the Université Cheikh Anta Diop’s (UCAD) sites located in the Roi Baudoin Hospital in Guédiawaye from June 2012 to March 2013. Guédiawaye is a small town situated on the coastal shoreline in the Dakar region, bordered to the East and the South by the town of Pikine and to the West by the city of Dakar, specifically by the municipal district of Parcelles Assainies [20]. It is located approximately 10 km from the capital, and covers an area of 30 km^2^. Its population is estimated at 452,168 inhabitants and the average annual rainfall amounts to approximately 600 mm. Over the past few years, malaria transmission has reached record levels in all 5 municipal districts within the *Départment*, namely Médina Gounas, Whakhinane Nimzath, Golf Sud, Sam Notaire and Ndiareme Limamoulaye. There are several reasons that account for the resurgence of malaria in the Dakar suburbs: (i) a high population density, (ii) cyclic flooding in lowland areas since the year 2000, (iii) the lack of a drainage system, and (iv) the existence of several wetland areas (containment basins, marshland, etc.)

#### Study design and interventions

The study was reviewed and approved by the “*Conseil National d’Ethique pour la Recherche en Santé”* (CNERS) of Senegal as well as the “Direction de la Pharmacie et des Médicaments” (DPM), the Senegalese regulatory authority, and Oxford Tropical Research Ethics Committee (OXTREC). An independent Data Safety Monitoring Board (DSMB) provided oversight for the study. An on-site Local Safety Monitor (LSM) acted as liaison between the site and the DSMB. The study was conducted in accordance with the Helsinki Declaration of 1964 (revised in 1996) and in accordance with Good Clinical Practice (Directive 2001/83/EC, amended 2003/63/EC). The University of Oxford acted as study sponsor. The trial was registered at the Pan-African Clinical Trial Registry (Ref No: PACTR 201-303-000-499-409); http://www.pactr.org/ and ClinicalTrials.gov (Ref No: NCT01658696); http://clinicaltrials.gov.

We conducted a randomised, controlled, single-blinded phase IIb efficacy trial. An independent statistician based at the University of Oxford provided sealed randomisation envelopes to the study site. Participants, fieldworker and laboratory analysts were blinded to study allocation of participants and remained blinded until after analysis was completed. The recombinant vectors have been previously described in detail [21]. Eligible study participants were randomised to receive either the ChAd63 ME-TRAP (5x10^5^ vp) as prime vaccination, followed eight weeks later by MVA ME-TRAP (2x10^8^ pfu) as booster or two doses of anti-rabies vaccine (0.5ml) as comparator at the same interval. All vaccines were administered intramuscularly.

#### Trial participants

Healthy men aged 18–50 years old were screened and 120 eligible volunteers were selected to participate in this trial. Prior to the commencement of the study, local community sensitisation (collective and individual) meetings were held to introduce to members of the community the purpose and nature of the clinical trial. Screening of eligible volunteers commenced after signed informed consent was obtained. Volunteers enrolled into the study were required to be in good health, within the age range, and residing in the study area for the duration of the study. Exclusion of volunteers occurred if there was any physical, haematological or biochemical evidence of ill health. In order to maintain confidentiality, anonymised HIV test were conducted using the standard rapid diagnostic kits and testing algorithm used by the Senegalese Ministry of Health and Prevention. A trained counsellor provided pre-test counseling. Eligible volunteers who participated in the trial met all of the inclusion criteria and none of the exclusion criteria.

#### Assessment of safety and reactogenicity

Volunteers were observed for at least 30 minutes after each vaccination. A number of volunteers were assigned to each of the 12 field workers who visited them at home on day one post-vaccination and were assessed in the clinic on day 14 and 7 after the first and second vaccinations respectively. The local solicited adverse events (AEs) were; pain, discoloration, swelling, warmth, itch, scaling or blistering at the injection site. Systemic solicited AEs were; headache, malaise, arthralgia, reported fever, nausea, myalgia and documented fever (temperature ≥37.5°C). Using pre-determined criteria, the intensity of AEs was graded as mild, moderate or severe. Serious adverse events (SAEs) were recorded during the whole study period. Laboratory abnormalities, recorded as adverse events were assessed by full blood count, creatinine and alanine aminotransferase.

#### Drug Treatment prior to PCR monitoring for P. falciparum infection

We administered a three-day course of directly observed atovaquone/proguanil (250mg/100mg) and artesunate (250mg) 7 days after the final vaccination (Days 63–65) in order to clear parasitaemia prior to PCR monitoring for new *P*. *falciparum* infections.

#### Immunogenicity

Immunogenicity to the TRAP antigen was assessed by IgG ELISA and *ex-vivo* interferon-gamma (IFNγ) ELISpot. The immunogenicity was determined at D0, D14 (2 weeks post vaccination with ChAd63 ME-TRAP), D63 (7 days post vaccination with MVA-ME-TRAP) and D161. While ME-TRAP ELISpots were performed on samples from all volunteers at D63 (the peak time-point for vaccine-induced cellular immunity), due to the high cost and staff requirement of the immunology analysis, we did a second randomization to allocate measurement of ELISpot responses at D0, D14 and D161 to 30 volunteers only. Fresh PBMCs were isolated from 40 ml of heparinized blood using Lymphoprep (Axis-Shield Diagnostics Ltd). ELISA and ELISpot assays were performed as previously described [17]. An AID ELISPOT plate reader with software v7 was used to count the spots. The mean of the negative control wells, containing cells and medium only, was subtracted from the mean of all the other duplicate wells for each volunteer. The mean response of all ME-TRAP peptide pool wells were then summed and expressed as SFC per million PBMC. Peptide pools are described in S3 Table. The maximum permitted value of the negative control well was 80 SFC, while the minimum for the positive well was 800 SFC. Responses were deemed positive if they were greater than the mean plus 3 standard deviations of the negative control wells of all assays performed in the study, which was 62 SFC.

Neutralising antibodies to the ChAd63 vector were assayed as previously described [17], on a separately recruited cohort of 205 adults living in the Keur Soce area of Dakar.

#### Efficacy

Efficacy was assessed by time to detection of malaria parasites in blood by quantitative PCR (qPCR) assays. Volunteers were followed up initially for 4 weeks by three times weekly blood sampling (0.5mls) for qPCR monitoring. Subsequently blood sampling was done once weekly for a further 4 weeks. Prior to the onset of the qPCR monitoring period, drug treatment was administered to volunteers to clear parasitaemia. Follow-up began 2 weeks after the second and final vaccination and 7 days after drug treatment. Volunteers diagnosed with malaria infection during follow-up were treated according to national guidelines.

#### qPCR Monitoring

qPCR monitoring and assays were conducted as previously described [13,16,18,19,22]. In summary, blood samples were collected on days 0 and 63 for baseline parasitaemia and during the efficacy follow-up period, blood was collected three times per week from day 70 to day 95 and once per week from day 98 to day 119. Blood samples were filtered with custom Whatman 24 well VFE plates and washed with PBS to remove white cells before DNA extraction using a modified QIAamp Blood Mini Kit protocol. DNA was extracted from 0.5ml filtered blood and eluted into 50 μl, from which 15 μl (i.e. 5 μl in triplicate) was amplified by quantitative PCR using primers (forward- 5’ GTAATTGGAATGATAGGAATTTACAAGGT 3’, reverse- 5’ TCAACTACGAACGTTTTAACTGCAAC 3’) and a TaqMan® probe (5′-FAM-AACAATTGGAGGGCAAG-NFQ-MGB-3′) specific for the multicopy 18S ribosomal RNA genes. We used an Applied Biosystems 7300 real time PCR system with quantification by Applied Biosystems 7300 system software v1.4 at UCAD. Similar further analysis of DNA and repeat QIAamp DNA extractions of frozen filtered blood samples were performed in Oxford using an Applied Biosystems Step One Plus real time PCR system, analysed using Step One Plus software v2.3. A pre-planned protocol-specified meta-analysis was conducted that combined the results of the Kenyan and Senegalese studies to give an overall estimate of efficacy across the two cohorts.

#### Statistical Analysis

The DSMB, sponsor and investigators agreed an analysis plan before unblinding. The primary efficacy analysis was the hazard ratio for vaccination as determined by Cox regression model for first PCR positive blood sample post-vaccination (1972. Regression models and life tables. J. R. Stat. Soc. 34: 187–220). The secondary efficacy analysis was the hazard ratio for vaccination, again by Cox regression for first PCR positive blood sample above a threshold of 10 parasites per ml. The time at risk was considered to begin one week after the final vaccination with MVA ME-TRAP. All analyses are presented unadjusted and then adjusted for potential confounders (age and bednet use). Safety data is presented for the ITT cohort (i.e. including all randomized participants), and immunogenicity and efficacy data is presented for the ATP cohort (i.e. excluding 3 subjects who did not complete their vaccination course).

Analysis was done using Stata version 11 (Statacorp, College Station, Texas).

Differences between immunological responses were assessed by Kruskal Wallis tests.

#### Sample size determination

We expected about 70% of adults to be PCR positive during follow up, thus providing 90% power to detect an efficacy of 50% and 80% power to detect an efficacy of 40% with 100 volunteers. The sample size was increased to 120 to allow for loss to follow up.

## Results

### Participant baseline characteristics and trial profile

**Fig 1:**
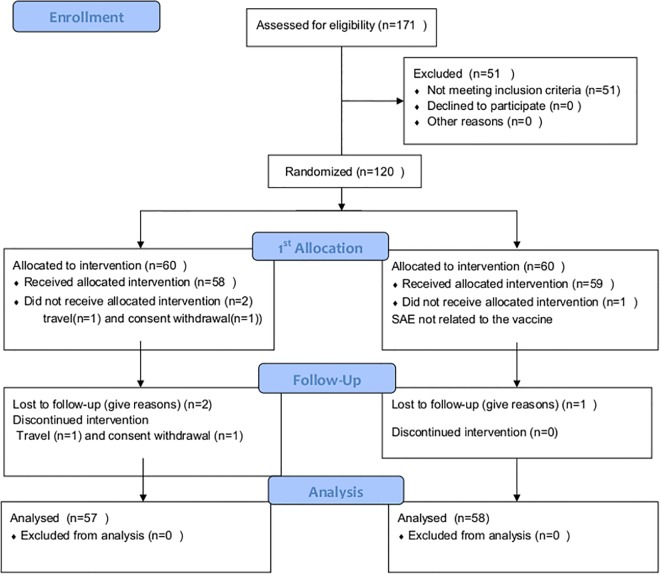
Consort 2010 flow diagram

One hundred and seventy one people were screened prior to randomisation and enrollment of 120 participants. The most common reasons for exclusion were positive hepatitis B (HBsAg) serology (n = 21), isolated hematology and or biochemistry abnormalities (n = 16) and abnormalities at the clinical examination (n = 13). One participant travelled to outside the study area before treatment allocation and was loss to follow up. No positivity to HIV was found. The participant flow is summarised in Fig 1 and shows an excellent completion rate in both arms of the study. The study was conducted within the populations living in the 5 districts of Guédiawaye. These five districts were stratified in low, moderate and high transmission areas. The participants were comparable for age, bednet use and local area of residence (Table 1).

**Table 1:** Baseline characteristics

	**TRAP group (n = 60)**		**Control group (n = 60)**		
**Parameter or category**	Mean/ Freq	% or IQR	Mean/ Freq	% or IQR	P
**Mean Age (Years)**	30.2	28.1 to 32.3	28.4	26.3 to 30.4	0.64
**Bed net use**					0.18
**yes**	5	8.3%	10	16.7%	
**No**	55	91.7%	50	83.3%	
**Location of residence**					0.21
**Wakhinane Nimzatt & Ndiareme limamoulaye**	19	31.7%	18	30%	
**Sam notaire**	12	20.0%	11	18.3%	
**Medina Gounass**	17	28.3%	17	28.3%	
**Golf Sud**	12	20.0%	14	23.3	

### Safety

No serious adverse event (SAE) related to vaccination was reported during the study period. One SAE was observed in the control group. The volunteer reported to the outpatient ward with fever, headache, chills, articulations pains, asthenia and dizziness and typhoid fever was diagnosed following examination and investigation. The volunteer was withdrawn from the study on September 2012.

**Fig 2:**
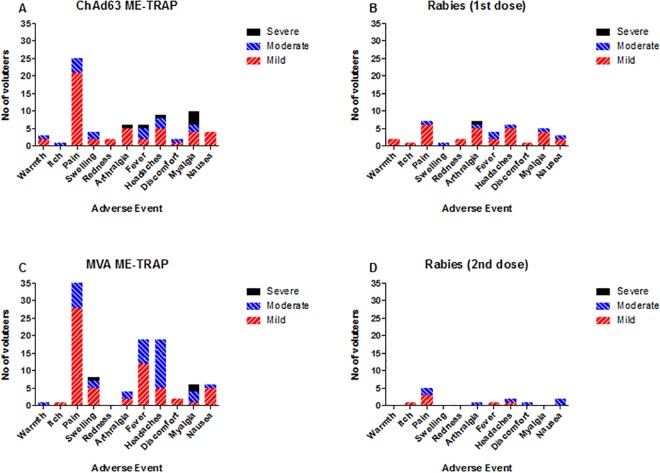
Local and systemic adverse event profiles for each vaccination, stratified by severity.

Pain, itching, redness, swelling and warmth were the most common local adverse events associated with the administration of the ChAd63 ME-TRAP vaccine Fig 2 with most reported as mild and some moderate in intensity. Grading criteria were defined in the clinical trial protocol and severity assessment criteria are also described in S1 and S2 Tables. The most common systemic adverse events post-ChAd63 ME-TRAP vaccination were fever, myalgia, discomfort, headache, arthralgia and nausea mostly mild to moderate with a few severe occurrences. As previously observed, the MVA ME-TRAP vaccine was more reactogenic than the ChAd63 ME-TRAP vaccine but was still well tolerated with the majority of AEs being mild in intensity. Similarly, the most common solicited local adverse events reported post-MVA ME- TRAP vaccination were swelling, pain, itching and warmth which lasted for between a few hours to 2 days. Systemic AEs reported post-MVA ME-TRAP were arthralgia, fever, headache, myalgia, nausea and discomfort. All unsolicited adverse events seen within 30 days post-vaccination were not vaccine related and were mild to moderate in nature.

### Clinical malaria cases

With regard to malaria incidence, 5 cases of clinical malaria occurred before the boost vaccination mostly in the malaria vaccine arm and 6 clinical malaria episodes after the boost vaccination, 4 of which were in the control group. A very low number of clinical malaria cases were recorded probably due to the unusually low malaria transmission in the year of the study. In previous years, higher seasonal transmission had been observed [23].

### Immunogenicity

**Fig 3:**
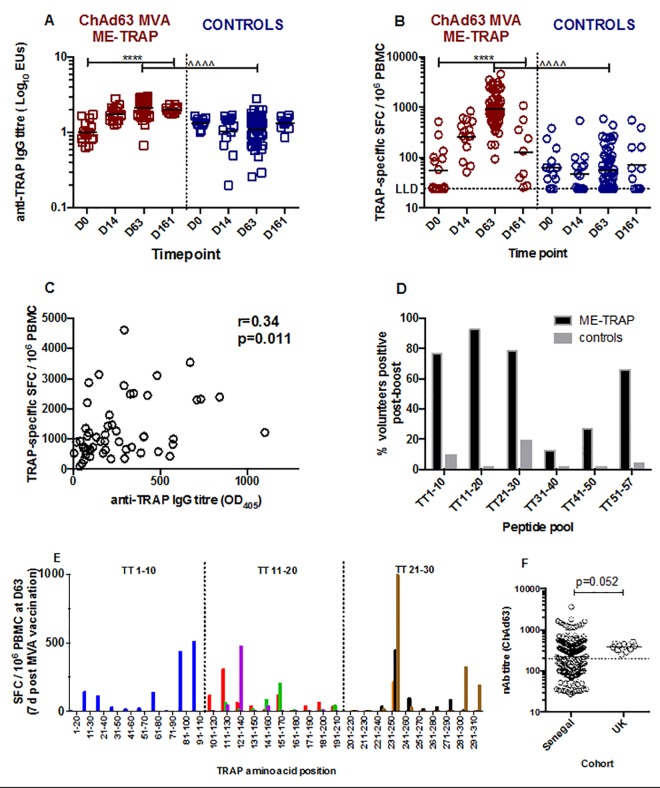
Vaccine immunogenicity. (A and B). Anti-TRAP IgG titres and IFN-γ ELISpot responses to TRAP peptide pools before and after vaccination. **** p<0.0001, Kruskall-Wallis with Dunn’s post-test; ^^^^ p<0.0001, 2-tailed Mann Whitney test. Lines represent geometric means. (C). Correlation between humoral and cellular immunogenicity for ME-TRAP vaccinees, 2-tailed Pearson’s test, n = 54. (D). Proportion of vaccinees with positive responses to TRAP peptide pools after boosting with MVA. Labels on x-axis refer to peptide pools described in S3 Table. E. Epitope mapping of TRAP peptide TT1-10, 11–20 and 21–30 using cryopreserved PBMC. Each colour represents a different volunteer, n = 6. F. Neutralising antibody titres to the ChAd63 vector measured in Senegalese and UK volunteers, 2-tailed Mann-Whitney.

Increases in anti-TRAP IgG responses were detected after priming with ChAd63 (log_10_ geometric mean titre (LGMT) 1.76 CI (1.5–2.0) and boosting with MVA in the ME-TRAP vaccinated group (LGMT 2.2, CI 2.0–2.3, p<0.0001 Kruskall-Wallis test, Fig 3A). No increase in anti-TRAP IgG responses were detected after either vaccination in control group, p = 0.3, Kruskall-Wallis test. At the peak of the vaccine-induced humoral response, titres were low, but comparable in magnitude to those observed in adults from the UK and The Gambia (unpublished data).

The priming vaccination with ChAd63 ME-TRAP induced detectable TRAP-specific T cells 14 days later as measured by ELISpot, at a geometric mean of 261 SFC per million PBMC (95% confidence interval (CI) 165–412) compared with 48 SFC (CI 30–79 SFC) among control vaccinees (p<0.001, Mann Whitney test, Fig 3B). Highest immunogenicity was identified 7 days after boosting vaccination with MVA ME-TRAP, at a geometric mean of 932 SFC (CI 754–1152) compared with 57 SFC per million (95% CI 44–72) among control vaccinees, p<0.0001 Mann Whitney test). The difference between groups was no longer significant 105 days after MVA ME-TRAP (129 SFC per million PBMC, CI 44–376 compared with 72 spots per million, 95%CI 33–158, p = 0.3).

There was a positive correlation between humoral and cellular immunogenicity among ME-TRAP vaccinees (Fig 3C, Pearson’s r = 0.34, p = 0.01). As previously observed with this antigen, TRAP peptide pools 1, 2, 3 and 6 were most frequently recognized with 66–93% of TRAP-vaccinated volunteers showing a positive response to these pools at the peak time point after MVA (Fig 3D). Nineteen percent of vaccinees in the control group had a positive response to pool 3 and ten percent to pool 1; few responses were detected to other pools. Epitope mapping with individual 20mer peptides was performed for pools 1 to 3 in a small number of volunteers to identify immunodominant peptides within pools. Responses to at least four peptides in each pool were detected, although some were to adjacent peptides suggesting epitopes spanned by two overlapping peptides (Fig 3E).

Neutralising antibodies to the ChAd63 vector, assayed in a separate Senegalese cohort, were detectable with LGMT of 216 (95% CI 188–247), with 56% of responses above the clinically relevant threshold of 200. In contrast, in a smaller cohort of UK adults [16], responses were higher (LGMT 370, 95% CI 330–436, p = 0.052, 2-tailed Mann Whitney test, Fig 3F) with 100% of responses above 200.

### Efficacy

**Fig 4:**
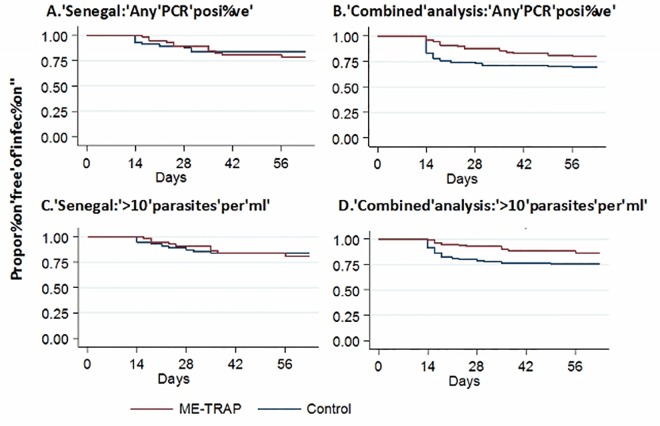
Kaplan-Meier plots of time to first infection. (A) First episode of PCR positivity at any threshold for the trial in Senegal. (B). Combined analysis of data from the Senegal and Kenya trials. (C). First episode of PCR positivity at >10 parasites per ml for the trial in Senegal. (D). Combined analysis of data from the Senegal and Kenya trials

Kaplan Meier plots of the distribution of time to PCR positivity are shown in Fig 4. Twelve of 57 ME-TRAP vaccinees became PCR positive during the intensive monitoring period as compared to 13 of the 58 controls. Using Cox regression analysis, efficacy was 8% but was not statistically significant (95% CI -100% to 59%), p = 0.8; Table 2 and Fig 4A). The secondary endpoint of PCR positivity (over 10 parasites per ml) also did not show significant protective efficacy (Fig 4C). There were 11 cases of malaria in the ME-TRAP vaccine arm as compared to 12 in the rabies arm resulting in an unadjusted efficacy of 9% (95% CI -100% to 60%). Efficacy was not affected by adjustment for covariates (Table 2) and remained non-significant. The hazard ratios for covariates for the primary endpoint of infection by PCR were long lasting insecticide-treated bednet use (LLIN) 1.66 (95%CI 0.57–4.8 p = 0.53); and age (per year) 1.01 (95%CI 0.97–1.06 p = 0.5).

**Table 2:** Vaccine efficacy by Cox regression for any parasitaemia and parasitaemia >10 per ml

** **	**TRAP**		**Control**		**Unadjusted Efficacy**		**Adjusted Efficacy**	
** **	N	n	N	n	Efficacy (95% CI)	p	Efficacy (95% CI)	p
**Any PCR positivity**	57	12	58	13	8% (-200–50%)	0.6	8% (-164–39%)	0.53
**>10 parasites/ml**	57	11	58	12	9% (-180–50%)	0.8	9% (-141–46%)	0.74

### Combined analysis of efficacy with analogous study in Kenya

**Fig 5:**
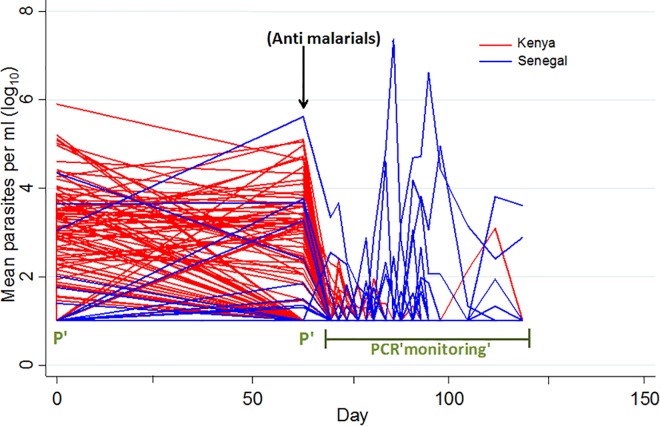
Parasite density as measured by PCR for each trial. Each line represents an individual volunteer. P = PCR testing performed. During the PCR monitoring follow-up period, blood samples were tested by PCR three times a week from day 70 to day 95 and once per week from day 98 to day 119.

Although the study in Senegal was underpowered to detect vaccine efficacy due to the unexpectedly low number of infections during the follow-up period, a protocol-specified meta-analysis after pooling the data of the Kenyan and Senegalese trials showed significant protective efficacy of 50% (95% CI 17%-70%; Table 3; Fig 4B and 4D). The difference in transmission dynamics between the two study sites is demonstrated in Fig 5, showing parasite density for each volunteer during the study. Volunteers in the Kenyan study that became infected became positive only during the first two weeks of PCR monitoring, in contrast to the study in Senegal where infections were detected throughout the follow-up period. In addition, parasite densities tended to be much higher in the Senegalese trial.

**Table 3:** Pooled vaccine efficacy by Cox regression for the studies in Kenya and Senegal.

** **	**TRAP**		**Control**		Unadjusted Efficacy*	
** **	N	n	N	n	Efficacy (95% CI)	p
**Any PCR positivity**	117	23	117	41	50% (17%-70%)	0.009
**>10 parasites/ml**	117	15	117	31	57% (19%-77%)	0.009

*Analysis of efficacy with adjustment for covariates was not performed as effect of age and bednet use varied between the two sites.

## Discussion

This clinical trial assessed the efficacy of ChAd63-MVA ME-TRAP prime-boost vaccination against *P*. *falciparum* infection in Senegalese adults in Guediawaye a suburb of the capital city Dakar. The study achieved a good completion rate with 114 volunteers out of 120 completing the trial and no vaccine-related serious adverse events were observed. The adverse events reported were mostly mild to moderate, in line with data from previous studies [13,14,21,24] and overall ChAd63-MVA ME-TRAP was found to be well tolerated.

ELISpot responses to TRAP peptides were detected at all-time points (baseline, day14, day 63 and day 161) in both groups, with baseline responses comparable to those observed in previous studies in Kenyan, Gambian and UK adults. [17] As expected, T cell immunogenicity in these malaria-exposed young Senegalese men peaked at one week after the MVA booster dose, at mean level of 923 SFC per million PBMCs. As previously observed in both malaria-naïve and semi-immune vaccinees, immunisation with the ME-TRAP construct induced responses to several epitopes spanning the length of the antigen, indicating that peptides are recognized through a number of different MHC alleles. These responses were however lower than those reported in Kenya at each time point, where the immune response peaked at 1421 SFC [19], as well as lower than in UK studies where the mean response exceeded 2000 [4].

The surprising difference in immunogenicity found between Kenya and Senegal may partially be explained by several reasons including the lower immunogenicity at baseline in Senegal (60 SFC in Guédiawaye compared with 112 SFC in Kilifi) The ChAd63 ME-TRAP priming in Kilifi may have acted as a booster dose to naturally acquired immunity. The absence of malaria exposure in the first weeks of follow up in Senegal may have pushed the rate ratio towards 1 and obscured any genuine protective efficacy. In other words, with the absence of malaria risk in both study groups in the first two weeks of the trial, the rate ratio at day 14 was still 1 and vaccine efficacy (1-RR) = 0. This period of zero vaccine efficacy pulls down the overall efficacy for all eight weeks. Malaria transmission is nearly perennial in Kenya with two different transmission seasons, while the transmission pattern in Senegal is very different. There is a long dry (minimal transmission) season of about 9 months followed by a wet season of 3 months. Malaria is actually transmitted only during part of the wet season. Transmission is therefore intense and short. Outside this transmission window, there may be a total absence of exposure.

Among 205 adults from another Senegalese population (Keur Socé in Central Senegal), serum samples tested for anti-ChAd63 neutralizing antibodies indicated that all had previous exposure to ChAd63 or a related adenovirus. Such results have been observed in other populations [25] and are most likely due to varying degrees of cross reactivity with related adenoviruses to which volunteers had been exposed. Importantly, this relatively low level of anti-vector ‘s immunity, is found even in European populations (i.e. UK) and have been shown not to associate with any reduced immunogenicity of the ChAd63 ME-TRAP vector [17–21], most likely because the dose of ChAd63 used is sufficient to overcome the modest levels of neutralizing antibodies to ChAd vectors found in unimmunized humans. A previous malaria efficacy study evaluating a DNA prime and human adenovirus serotype 5 (AdHu5) vector for boosting did demonstrate a modest effect of pre-existing anti-vector immunogenicity on ELISA responses to the AMA1 antigen and trends for association with ELISPOT responses to CSP and AMA1[26]. None of the participants in this study that had high anti-AdHu5 neutralising antibodies prior to vaccination were protected against CHMI; however there was no statistical association between anti-vector immunity and efficacy. Therefore, the low prevalence of serum ChAd63-neutralizing antibodies in Guédiawaye and the excellent immune responses generated even in the presence of detectable *in vitro* vector-neutralizing immunity [27–30] make ChAd63 a promising vaccine vector.

Vaccine efficacy against the primary endpoint (parasitaemia>0) was significantly different than that observed in the parallel study in Kenya where a vaccine efficacy of 67% was observed. A pre-specified pooled analysis of the data from both studies using Cox regression showed an overall vaccine efficacy in the two populations of 50% (95% confidence interval 17–70%, p = 0.009) for time to first PCR positivity.

Although the unadjusted confidence intervals of the efficacy estimates in the two sites were overlapping, there was a significant interaction between the effects of site and vaccination (p = 0.009) but not with time since vaccination (p = 0.13). This provides statistical evidence from pooled Senegalese and Kenya datasets that there was variation in efficacy between the two studies rather than variation in efficacy over time since vaccination.

Several parasitological differences between the two sites that may be relevant to the apparent different vaccine efficacy are noteworthy:

1. PCR positivity was more prevalent before monitoring commenced in Kenya compared to Senegal;
2. After antimalarial treatment, the incidence of PCR positivity was higher in Kenya yielding more power to detect efficacy than in the Senegalese study.
3. The PCR data showed higher density parasitaemia in the trial in Senegal compared with the one in Kilifi, Kenya, representing a potentially more stringent challenge for the vaccine in the presence of less background malaria immunity. However, this likely reflects lower malaria transmission in the Senegalese population where the study was done, so that bites are less frequent and PCR positivity is less frequent; but when an infection occurs because prior exposure has been lower there is less blood stage immunity and therefore a higher peak parasitaemia is reached. The PCRs became positive in Kilifi only for a two-week period after anti-malarial treatment with no positives appearing later. In our Senegal study in contrast, the emergence of infections was evenly distributed throughout the follow-up period so that if vaccine efficacy falls very quickly lower efficacy could be seen in Senegal. However, the lack of significant interaction between site and time since vaccination in the combined model (above, p = 0.13) does not support this possibility.

It should be noted that in both sites examples of brief PCR positivity, without sustained parasite growth was observed, likely reflecting blood-stage immunity. Microscopy along with the qPCR was not performed after Atovaquone/Proguanil treatment.

An alternative explanation for the lack of vaccine efficacy in Senegal simply relates to lower vaccine immunogenicity. In UK CHMI studies a level of > 2000 SFU / million PBMCs was induced consistently by the ChAd-MVA regime [15–21] and this was associated with significant efficacy that correlated with T cell numbers. Notably the ChAd63 vaccine alone induced about 864 SFU / million PBMCs and no sterile protection or delay to patency was observed. The Kenyan response level was a geometric mean of 1694 SFU, closer to the UK ChAd63-MVA level. Hence, the reduction in T cell immunogenicity in Senegal to levels close to that seen with the non-protective ChAd63 only vaccination in the UK might in part explain lower efficacy. If so, this would highlight the need to use vaccine regimes and doses that consistently induce high-level T cell responses in every population.

In addition to enhancing cellular immune responses to TRAP, vaccine efficacy might potentially be improved by including additional liver-stage antigens, such as PfLSA1 and PfLSAP2. These antigens have recently been shown to induce sterile efficacy against transgenic parasites in a murine model of malaria and are progressing to clinical evaluation [31]. Another promising strategy is a combination approach harnessing the ability of viral vectors to induce potent cellular immunity in combination with a protein-in-adjuvant approach to inducing high antibody titres. We have recently reported high efficacy of such an approach combining the leading malaria vaccine candidate RTS,S with ChAd63 and MVA ME-TRAP [32].

Overall, the results of safety, immunogenicity and efficacy of the ChAd63-MVA vaccination strategy in Kenyan and Senegalese young men gave encouraging results and provide, to our knowledge, the first indication of potential field efficacy of any vaccine designed to induce protective cellular immunity. It is now important that protective efficacy be assessed in younger populations, especially young children and infants, and these trials are underway.

## Supporting Information

S1 Table Criteria used to determine grade of solicited local adverse events. (PDF)

S2 Table Criteria used to Assessment of Causality of Adverse Events. (PDF)

S3 Table Peptide Pooling Scheme used for ELISPOT Assays (PDF)

S1 Fig Trial profile (PDF)

S1 Appendix Protocol (PDF)

S2 Appendix Consort 2010 Checklist (PDF)

S3 Appendix Malaria positivity dataset. (PDF)
