# Supplementary material for: Safety, Immunogenicity and Efficacy of Prime-Boost Vaccination with ChAd63 and MVA Encoding ME-TRAP against Plasmodium falciparum Infection in Adults in Senegal
Source: PLoS One. 2016 Dec 15;11(12):e0167951. doi: 10.1371/journal.pone.0167951 (PMC5158312; doi:10.1371/journal.pone.0167951)
Supplement: S1 Table — (PDF) [file pone.0167951.s001.pdf]

S1 Table: Criteria used to determine grade of solicited local adverse events.

| Solicited Adverse Event | Parameter Assessed | Grade | Definition |
|---|---|---|---|
| Discoloration | Diameter | Not Present | 0 mm |
| | | Mild | < 50mm |
| | | Moderate | 50 – 100mm |
| | | Severe | > 100mm |
| Swelling | Diameter | Not Present | 0 mm |
| | | Mild | < 20mm |
| | | Moderate | 20 – 50mm |
| | | Severe | > 50mm |
| Pain | Function | Not Present | No pain at all |
| | | Mild | Painful to touch, no restriction in movement of arms, able to work, drive, carry heavy objects as normal |
| | | Moderate | Painful when limb is moved (i.e. restriction in range of movement in arm, difficulty in carrying objects) |
| | | Severe | Severe pain at rest (i.e. unable to use arm due to pain.) |
| Systemic Aes | Function | Not Present | Absence of the indicated symptom |
| | | Mild | Awareness of a symptom but the symptom is easily tolerated |
| | | Moderate | Discomfort enough to cause interference with usual activity |
| | | Severe | Incapacitating; unable to perform usual activities; requires absenteeism or bed rest |
