# Supplementary material for: Safety, Immunogenicity and Efficacy of Prime-Boost Vaccination with ChAd63 and MVA Encoding ME-TRAP against Plasmodium falciparum Infection in Adults in Senegal
Source: PLoS One. 2016 Dec 15;11(12):e0167951. doi: 10.1371/journal.pone.0167951 (PMC5158312; doi:10.1371/journal.pone.0167951)
Supplement: S2 Table — (PDF) [file pone.0167951.s002.pdf]

S2 Table: Criteria used to Assessment of Causality of Adverse Events.

No Relationship	No temporal relationship to vaccination *and*<br>Alternate aetiology (clinical state, environmental or other interventions); *and*<br>Does not follow known pattern of response to study product
Possible	Reasonable temporal relationship to vaccination ; *or*<br>Event not readily produced by clinical state, environmental or other interventions; *or*<br>Similar pattern of response to that seen with other vaccines
Probable	Reasonable temporal relationship to vaccination ; *and*<br>Event not readily produced by clinical state, environment, or other interventions *or*<br>Known pattern of response seen with other vaccines
Definite	Reasonable temporal relationship to vaccination ; *and*<br>Event not readily produced by clinical state, environment, or other interventions; *and*<br>Known pattern of response seen with other vaccines
