# Supplementary material for: Safety, Immunogenicity and Efficacy of Prime-Boost Vaccination with ChAd63 and MVA Encoding ME-TRAP against Plasmodium falciparum Infection in Adults in Senegal
Source: PLoS One. 2016 Dec 15;11(12):e0167951. doi: 10.1371/journal.pone.0167951 (PMC5158312; doi:10.1371/journal.pone.0167951)
Supplement: S3 Table — (PDF) [file pone.0167951.s003.pdf]

S3 Table: Peptide Pooling Scheme used for ELISPOT Assays

Peptide Name	Peptide sequence T9/96	Peptide sequence 3D7	T9/96 Peptide Pool	3D7 Peptide Pool
TRAP-1	MNHLGNVKYLVIVFLIFFDL		TT1-10	TD1-10
TRAP-2	VIVFLIFFDLFLVNGRDVQN		TT1-10	TD1-10
TRAP-3	FLVNGRDVQNNIVDEIKYSE	FLVNGRDVQNNIVDEIKYRE	TT1-10	TD1-10
TRAP-4	NIVDEIKYSEEVCNDQVDLY	NIVDEIKYREEVCNDEVDLY	TT1-10	TD1-10
TRAP-5	EVCNDQVDLYLLMDCSGSIR	EVCNDEVDLYLLMDCSGSIR	TT1-10	TD1-10
TRAP-6	LLMDCSGSIRRHNWVNHAVP		TT1-10	TD1-10
TRAP-7	RHNWVNHAVPLAMKLIQQLN		TT1-10	TD1-10
TRAP-8	LAMKLIQQLNLNDNAIHLYV	LAMKLIQQLNLNDNAIHLYA	TT1-10	TD1-10
TRAP-9	LNDNAIHLYVNVFSNNAKEI	LNDNAIHLYASVFSNNAREI	TT1-10	TD1-10
TRAP-10	LNDNAIHLYVNVFSNNAKEI	SVFSNNAREIIRLHSDASKN	TT1-10	TD1-10
TRAP-11	IRLHSDASKNKEKALIIIRS	IRLHSDASKNKEKALIIIKS	TT11-20	TD11-20
TRAP-12	KEKALIIIRSLLSTNLPYGR	KEKALIIIKSLLSTNLPYGK	TT11-20	TD11-20
TRAP-13	LLSTNLPYGRTNLTDALLQV	LLSTNLPYGKTNLTDALLQV	TT11-20	TD11-20
TRAP-14	TNLTDALLQVRKHLNDRINR		TT11-20	TD11-20
TRAP-15	RKHLNDRINRENANQLVVIL		TT11-20	TD11-20
TRAP-16	ENANQLVVILTDGIPDSIQD		TT11-20	TD11-20
TRAP-17	TDGIPDSIQDSLKESRKLSD		TT11-20	TD11-20
TRAP-18	SLKESRKLSDRGVKIAVFGI		TT11-20	TD11-20
TRAP-19	RGVKIAVFGIGQGINVAFNR		TT11-20	TD11-20
TRAP-20	GQGINVAFNRFLVGCHPSDG		TT11-20	TD11-20
TRAP-21	FLVGCHPSDGKCNLYADSAW		TT21-30	TD21-30
TRAP-22	KCNLYADSAWENVKNVIGPF		TT21-30	TD21-30
TRAP-23	ENVKNVIGPFMKAVCVEVEK		TT21-30	TD21-30
TRAP-24	MKAVCVEVEKTASCGVWDEW		TT21-30	TD21-30

TRAP-25	TASCGVWDEWSPCSVTCGKG		TT21-30	TD21-30
TRAP-26	SPCSVTCGKGTRSRKREILH		TT21-30	TD21-30
TRAP-27	TRSRKREILHEGC TSEIQEQ	TRSRKREILHEG CTSELQEQ	TT21-30	TD21-30
TRAP-28	EGCTSEIQEQCEEE RCPPKW	EGCTSELQEQCE EERCLPKR	TT21-30	TD21-30
TRAP-29	CEEERCPPKWEPL DVPDEPE	CEEERCLPKREP LDVPDEPE	TT21-30	TD21-30
TRAP-30	EPLDVPDEPEDDQPRPRGDN		TT21-30	TD21-30
TRAP-31	DDQPRPRGDNSSV QKPEENI	DDQPRPRGDNF AVEKPNENI	TT31-40	TD31-40
TRAP-32	SSVQKPEENIIDNN PQEPSP	FAVEKPNENIID NNPQEPSP	TT31-40	TD31-40
TRAP-33	IDNNPQEPSPNPEE GKDENP	IDNNPQEPSPNPE EGKGENP	TT31-40	TD31-40
TRAP-34	NPEEGKDENPNGF DLDENPE	NPEEGKGENPNG FDLDENPE	TT31-40	TD31-40
TRAP-35	NGFDLDENPENPP NPDIPEQ	NGFDLDENPENP PNPPNPPN	TT31-40	TD31-40
TRAP-36	NPPNPDIPEQKPNI PEDSEK	NPPNPPNPPNPP NPPNPPNP	TT31-40	TD31-40
TRAP-37	*NONE*	PPNPPNPPNPDIP EQKPNIP	TT31-40	TD31-40
TRAP-38	DIPEQKPNIPEDSE KEVPSD	DIPEQKPNIPEDS EKEVPSD	TT31-40	TD31-40
TRAP-39	EDSEKEVPSDVPKNPEDDRE		TT31-40	TD31-40
TRAP-40	VPKNPEDDREENFDIPKKPE		TT31-40	TD31-40
TRAP-41	ENFDIPKKPENKHDNQNNLP		TT41-50	TD41-50
TRAP-42	NKHDNQNNLPND KSDRNIPY	NKHDNQNNLPN DKSDRYIPY	TT41-50	TD41-50
TRAP-43	NDKSDRNIPYSPL PPKVLDN	NDKSDRYIPYSP LAPKVLDN	TT41-50	TD41-50
TRAP-44	SPLPPKVLDNERK QSDPQSQ	SPLAPKVLDNER KQSDPQSQ	TT41-50	TD41-50
TRAP-45	ERKQSDPQSQDNNGNRHVPN		TT41-50	TD41-50
TRAP-46	DNNGNRHVPNSEDRETRPHG		TT41-50	TD41-50
TRAP-47	SEDRETRPHGRNNENRSYNR		TT41-50	TD41-50
TRAP-48	RNNENRSYNRKYNDTPKHPE		TT41-50	TD41-50
TRAP-49	KYNDTPKHPEREEHEKPDNN		TT41-50	TD41-50
TRAP-50	REEHEKPDNNKK KGESDNKY	REEHEKPDNNKK KAGSDNKY	TT51-57	TD51-57
TRAP-51	KKKGESDNKYKI AGGIAGGL	KKKAGSDNKYKI AGGIAGGL	TT51-57	TD51-57

TRAP-52	KIAGGIAGGLALL ACAGLAY	KIAGGIAGGLALL ACAGLAY	TT51-57	TD51-57
TRAP-53	ALLACAGLAYKF VVPGAATP	ALLACAGLAYKF VVPGAATP	TT51-57	TD51-57
TRAP-54	KFVVPGAATPYA GEPAPFDE	KFVVPGAATPYA GEPAPFDE	TT51-57	TD51-57
TRAP-55	YAGEPAPFDETL GEEDKDLD	YAGEPAPFDETL GEEDKDLD	TT51-57	TD51-57
TRAP-56	TLGEEDKDLDEP EQFRLPEE	TLGEEDKDLDEP EQFRLPEE	TT51-57	TD51-57
TRAP-57	EPEQFRLPEENE WN	EPEQFRLPEENE WN	TT51-57	TD51-57
