# Supplementary figures and images for: Safety, Immunogenicity and Efficacy of Prime-Boost Vaccination with ChAd63 and MVA Encoding ME-TRAP against Plasmodium falciparum Infection in Adults in Senegal
Source: PLoS One. 2016 Dec 15;11(12):e0167951. doi: 10.1371/journal.pone.0167951 (PMC5158312; doi:10.1371/journal.pone.0167951)

S1Fig: Trial profile

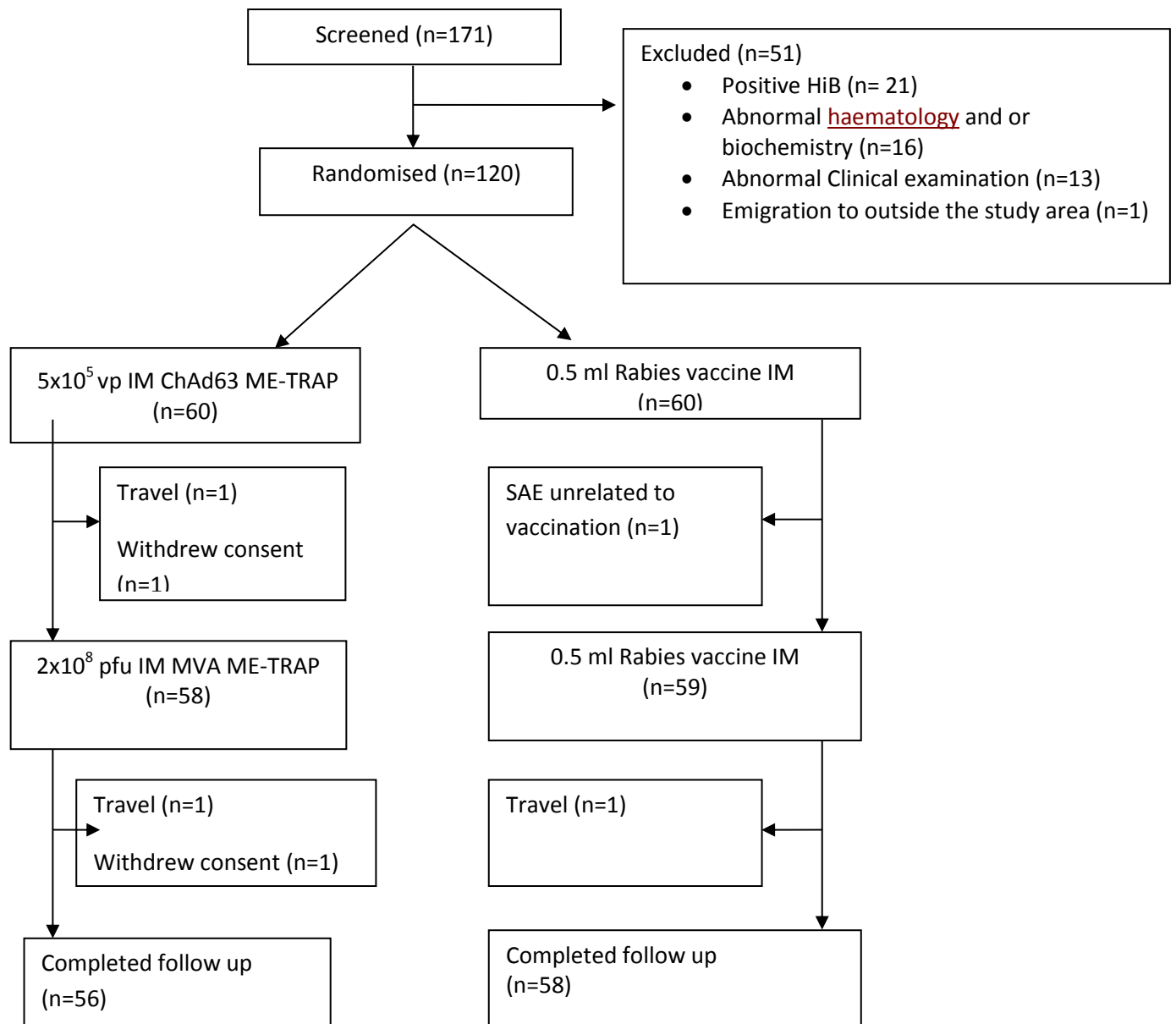

Supplement: S1 Fig — (PDF) [file pone.0167951.s004.pdf]
