# Supplementary material for: Safety, Immunogenicity and Efficacy of Prime-Boost Vaccination with ChAd63 and MVA Encoding ME-TRAP against Plasmodium falciparum Infection in Adults in Senegal
Source: PLoS One. 2016 Dec 15;11(12):e0167951. doi: 10.1371/journal.pone.0167951 (PMC5158312; doi:10.1371/journal.pone.0167951)
Supplement: S1 Appendix — (PDF) [file pone.0167951.s005.pdf]

1. TITLE OF PROJECT

Efficacy Study of ChAd63-MVA ME-TRAP prime-boost Vaccination against *Plasmodium falciparum* infection.

2. INVESTIGATORS

Senegal (University Cheikh Anta Diop, Dakar)

**Title/Name**	**Profession**	**Duties**	**Full-time equivalent**
Dr Badara Cisse[1]	Doctor	PI	33%
Prof. Babacar Faye[1]	Doctor	Co-PI	20%
Prof. Oumar Gaye[1]	Doctor	Scientific adviser	5%
Prof Tandakha Dieye[4]	Pharmacist	Investigator	10%
Dr Jean-Louis Ndiaye[1]	Doctor	Scientific adviser	5%
Dr Aly Gueye[1]	Doctor	Head of clinical operations	100%
Dr Massamba Syll[1]	Pharmacist	Quality control officer	75%
Victorine Mensah[1]	Scientific officer	Laboratory Immunology	100%
Amy Ndaw[1]	Laboratory Technician	Laboratory haematology, biochemistry, serology	100%
Maguette Ndiaye	Biologist	PCR	20%
Dr Annie Abiola[1]	Pharmacist	qPCR officer	50%
Dr Anna Beye	Pharmacist	Responsible for vaccine management	100%
Dr Souley Lelo[2]	Doctor	Researcher	50%
Jules-François Gomis[1]	IT technician	Database manager	50%
Dr Allé Baba Dieng[3]	Doctor	Internal monitor	15%
Prof. Cheikh T Ndour[3]	Doctor	Safety monitor	5%
Ms Taggy Danfakha[3]	Nurse	Post-vaccine follow-up and sample taking	100%
Mr Roger Senghor[3]	Nurse	Post-vaccine follow-up and sample taking	100%
Ms Kane[2]	Social worker	Liaison officer with the Guediawaye grass-roots community organizations to facilitate screening and recruitment of volunteers	25%
Ms Diallo[2]	Social worker	Facilitator – awareness-raising	
Dr Toure[2]	Hospital director	Facilitator	0%
Mr Mbaye Dia[2]	Nursing supervisor	Facilitator	0%
Abdoulaye Mbaye[1]	Driver	Driver/logistician	100%
Talla Diop[1]	Driver	Driver/mechanic	100%
Sassy Fall[1]	Data entry operator	Data entry	100%
Ndeye Marie Diop[1]	Data entry operator	Data entry	100%
Aminata Sow[1]	Data entry operator	Data entry	100%
Ciré Ly[1]	Chief accountant	Administration/Finance	50%
Michel Ndong[1]	Accounts assistant	Accounting	100%
Rokhaya Mbaye[1]	Executive assistant	Administrative assistant	100%

[1=]Laboratory of Medical Parasitology/UCAD
[2=]Roi Baudouin Hospital

[3]=Department of Infectious Diseases, Fann University Hospital
[4] = Laboratoire de Bactériologie Virologie de l'Hopital A Le Dantec

United Kingdom (Jenner Institute, Nuffield Dept. of Clinical Medicine, University of Oxford)

- Adrian Hill
- Susanne Sheehy
- Nicholas Anagnostou
- Alison Lawrie
- Rachel Roberts

3. ABBREVIATIONS

**AdHu**	Human Adenovirus
**AE**	Adverse event
**ALT**	Alanine transaminase
**AMA**	Apical Membrane Antigen
**ATP**	According to protocol
**ChAd63**	Chimpanzee adenovirus 63
**CRF**	Case Report Form
**CEF**	Chicken Embryo Fibroblasts
**CRFKS**	Centre de Recherche et Formation de Keur Soce
**CTF**	Clinical Trials Facility
**CTL**	Cytotoxic T-Lymphocytes
**CSP**	Circumsporozoite protein
**D**	Day
**DNA**	Deoxyribonucleic acid
**DSMB**	Data Safety and Monitoring Board
**ELISA**	Enzyme –linked immunosorbent assay
**ELISPOT**	Enzyme- linked immunospot
**EPI**	Expanded Programme on Immunisation
**ERC**	Ethical Review Committee
**FACS**	Fluorescence - activated cell sorter
**FBC**	Full Blood Count
**FP9**	Fowl pox 9
**GCP**	Good Clinical Practice
**GMP**	Good Manufacturing Practice
**HIV**	Human immunodeficiency virus
**HLA**	Human leukocyte antigen
**IB**	Investigator Brochure
**ICS**	Intracellular cytokine staining

**IFNγ**	Interferon- gamma
**IM**	Intramuscular
**IMP**	Investigational Medicinal Products
**ITN**	Insecticide treated net.
**ITT**	Intention to treat
**KSRC**	Keur Soce rural community
**LFT**	Liver Function Test
**LSM**	Local Safety Monitor
**ME- TRAP**	Multiple epitope string with thrombospondin- related adhesion protein
**MSP**	Merozoite surface protein
**MVA**	Modified vaccinia Virus Ankara
**OXTREC**	Oxford Tropical Research Ethics Committee
**PCR**	Polymerase Chain Reaction
**PI**	Principal Investigator
**Pfu**	Plaque forming units
**DPL**	Direction de la Pharmacie et des Laboratoires
**PBMC**	Peripheral Blood Mononuclear Cell
**SAE**	Serious Adverse Event
**SFC**	Spot Forming Cells
**SIV**	Simian Immunodeficiency Virus
**SOP**	Standard Operating Procedure
**TMF**	Trial master file
**TRAP**	Thrombospondin Related Adhesion Protein
**UCAD**	University Cheikh Anta Diop, Dakar
**VCT**	Voluntary Counselling and Testing
**Vp**	Viral particles

4. ABSTRACT

Malaria transmission is falling in some parts of Africa as bednets and anti-malarials become more widely available. However, transmission still persists and it appears that additional control measures are required. The leading malaria vaccine candidate in development is RTS,S which has efficacy against clinical malaria measured at 30-50% in the field. This partial protection might be enhanced by combination with other components. The other vaccination approach that has produced repeatable efficacy in humans is the use of viral vectors to induce T cell responses. Previous attempts with this vaccine approach have been effective in challenge studies in Oxford, but ineffective in the field, probably because of reduced immunogenicity.

Recently, studies in Oxford, Kenya and the Gambia have shown higher levels of immunogenicity by using a chimpanzee adenovirus (ChAd63) followed by an attenuated vaccinia virus (modified vaccinia Ankara) to deliver the pre-erythrocytic antigen, multiple epitope string with thrombospondin- related adhesion protein (ME-TRAP).The increase in immunogenicity has led to sterile protection in 3 out of 14 volunteers and partial protection in 5 out of 14 volunteers in challenge studies. It is important to determine efficacy before having larger trials in children.

We propose a Phase 2b study of 120 healthy adult men in Senegal. We will assess the efficacy and further evaluate the immunogenicity and safety profile of this vaccination strategy. We also intend to assess the correlates of efficacy and natural immunity.

5. BACKGROUND INFORMATION AND SCIENTIFIC RATIONALE

Introduction

Malaria transmission has been on the decline in some parts of Africa in association with scaling up of malaria control measures (1-7). This has led some to suggest the possibility that elimination is possible (8). This is desirable but ambitious: there were 500 million malaria episodes worldwide in 2002 and over a million deaths in Africa (9, 10). Previous attempts at elimination have had mixed success (11-13). It is currently thought that additional control measures will be required (14), including vaccination (15).

Progress towards a malaria vaccine

The candidate pre-erythrocytic malaria vaccine RTS,S targeting the circumsporozoite protein, is the most advanced vaccine in development. Efficacy with the AS01 adjuvant in 5 to 17 month old children was 52.9% (95%CI 28-69%) in Kenya and Tanzania over 8 months of follow up (16) and 45.8% over 15 months of follow up (17). A Phase III trial is now in progress. The observed efficacy of RTS,S is partial, indicating that we should continue to develop other candidate vaccines, which could be used in combination with RTS,S if efficacy is established, or

give rise to more efficacious approaches. The only other vaccination approach that has demonstrated partial efficacy in humans is using virally vectored vaccines to induce T cells.

Previous studies with T cell inducing malaria vaccines

The induction of T cells against malaria antigens can be maximized using a heterologous prime boost approach, where two different vectors are used sequentially, both delivering the same recombinant malaria antigen. We have focused on the multiple epitope string with thrombospondin- related adhesion protein (ME-TRAP) antigen, since it contains a high number of T cell epitopes with more limited polymorphism than the T cell epitope region of the circumsporozoite (CS) antigen. Furthermore T cell responses to thrombospondin- related adhesion protein (TRAP) are protective in mice and in humans.

The first such vaccination regimen to be subjected to field trials in Africa was deoxyribonucleic acid (DNA) ME-TRAP and modified vaccinia virus Ankara (MVA) ME-TRAP (18) in The Gambia, followed by fowl pox (FP9) ME-TRAP and MVA ME-TRAP in Kenya (19).

Neither of these vaccines were efficacious in the field, and the immunogenicity was lower than expected. Furthermore, FP9 ME-TRAP shows variability in potency by batch (20). Further development of T cell inducing vaccination in Oxford in pre-clinical studies has therefore examined more immunogenic priming vectors such as adenoviruses, in order to attain greater efficacy.

Clinical studies of chimpanzee adenovirus 63 **(**ChAd63 ME-TRAP) and MVA ME-TRAP

ChAd63 encoding ME-TRAP is more protective than either FP9 or MVA encoding ME-TRAP in animal studies (21, 22) and has to date been administered to 116 healthy volunteers in the UK, 46 healthy adult volunteers in Kenya and the Gambia and 24 healthy Gambian children (aged 2-6 years). Over 700 healthy volunteers (including more than 200 children) and 5 HIV positive volunteers have received MVA ME-TRAP in Oxford, Kenya and Gambia (19, 23-25). Both vaccines have been shown to be safe and well tolerated (see ChAd63 ME-TRAP & MVA ME-TRAP Investigator Brochures) (O'Hara et al. submitted). No linked serious adverse events have been reported for either vaccine.

The combination of priming with ChAd63 ME-TRAP and boosting with MVA ME-TRAP has been more immunogenic in humans than previous vaccinations (2400 T cells per million peripheral blood mononuclear cells (PBMCs) by enzyme- linked immunospot (ELISPOT) compared with less than 500 per million for DNA/MVA ME-TRAP or FP9/MVA ME-TRAP) and has demonstrated greater clinical efficacy in experimental challenge studies of volunteers in Oxford. In the UK clinical trial, MAL034, 3/14 volunteers demonstrated sterile protection (21%) and 5/14 demonstrated partial protection (36% - a delay in time to diagnosis) (Figure 1 A, Ewer et al submitted). Of note, on re-challenge 8 months later, all 3 sterilely protected volunteers demonstrated evidence of persisting protection, with 1 volunteer demonstrating sterile protection

and the other two partial protection. In this study, protection in these volunteers strongly correlated with levels of mono-functional CD8+ interferon- gamma (IFNγ) T cells (Figure 1 B). In this study all 12 unvaccinated control volunteers developed malaria, as have all the 88 unvaccinated control volunteers challenged to date in Oxford (19).

Time to polymerase chain reaction (PCR) positivity as an endpoint in field studies.

The data from sporozoite challenge with a laboratory strain of *P. falciparum* in non-immune volunteers are encouraging, but it is uncertain how well this model predicts vaccine efficacy in the field. Previous studies have relied on blood films to diagnose infection, but PCR based technology is considerably more sensitive (26-28). Building on experience of PCR monitoring of vaccinees and controls in recent sporozoite challenge studies in volunteers, a field efficacy study has been conducted in the Gambia using delay in time to PCR-measured parasitaemia as a study endpoint (29). Adult subjects were recruited, treated with curative anti-malarials, and then followed up by daily finger-prick blood testing to identify the first episode of PCR-measured parasitaemia. Subjects given anti-malarial prophylaxis were compared with control subjects, and were less frequently parasitaemic as measured by PCR.

There are few data with which to compare efficacy rates between the sporozoite challenge model and field studies. RTS, S/AS02, the most widely studied malaria vaccine, has consistently protected 30-45% of volunteers challenged experimentally with sporozoites about 2 weeks after final vaccination. However, efficacy of this vaccine against challenge with heterologous parasite strains in the field (The Gambia) gave about 70% protection during the first two months after vaccination using blood-film patent infection in semi-immune adults as the endpoint (30).

1 A

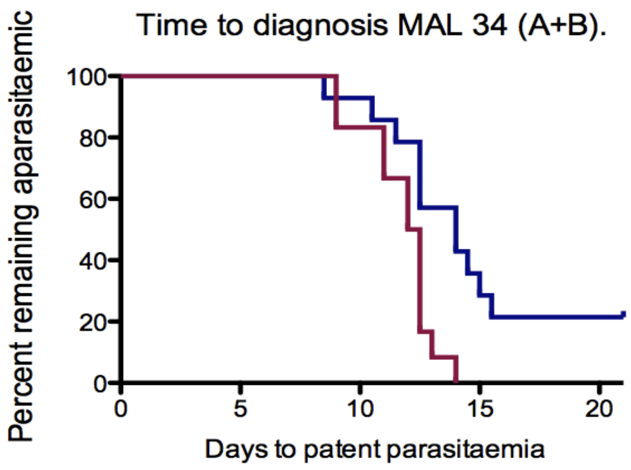

1B

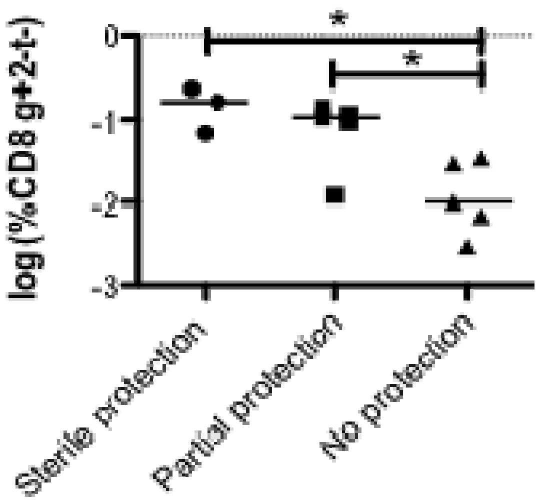

Figure 1: *Data from 14 healthy malaria naïve adult volunteers vaccinated with 5 x* 10^{10} *vp ChAd63 ME-TRAP intramuscularly, followed 8 weeks later by 2 x* 10^{8} *pfu MVA ME-TRAP intradermally.* ***Figure 1A:*** *Clinical efficacy on heterologous sporozoite challenge with 3D7 P. falciparum conducted in 2 phases. The 12 control volunteers (red line) were all diagnosed with malaria. 58% of vaccines (blue line) demonstrated clinical efficacy; 3/14 volunteers demonstrated sterile protection, 5/14 volunteers demonstrated partial protection (a delay in time to diagnosis).* ***Figure 1B:*** *Vaccinees with both sterile and partial protection had significantly higher mono-functional CD8+ interferon- gamma (IFNγ) T cell levels than non-protected vaccinees.*

Investigational products relevant to this application.

The Modified vaccinia Ankara (MVA) vector

The MVA vector was selected for its safety and immunogenicity profile. Vaccinia was successfully used to vaccinate against and eliminate smallpox. MVA was originally derived from the vaccinia strain Ankara by over 500 serial passages in primary chicken embryo fibroblasts (CEF cells). MVA has six major genomic deletions compared to the parental Ankara genome and cannot replicate in non-transformed mammalian cells. The viral genome has been proven to be stable through a large series of passages in chicken embryo fibroblasts (31). MVA shows no cytopathic effect or plaque formation in cells of human origin. In irradiated mice MVA did not elicit any morbidity or lethality even when administered at high doses intra-cerebrally (32). From 1972 until 1980 (the end of compulsory smallpox vaccination) MVA was licensed in Germany (33) and was included in the official immunisation schedule (34). In a large field study carried out in Germany over 120,000 previously unvaccinated individuals were vaccinated with MVA (0.2 ml) administered either intra-dermally or subcutaneously. The study population included high-risk groups (e.g. people suffering from allergies, elderly people, alcoholics) (31). MVA proved to be non-contagious and avirulent. Viral replication is blocked late during infection of cells but importantly viral and recombinant protein synthesis is unimpaired even during this abortive infection. Replication-deficient recombinant MVA has been viewed as an exceptionally safe viral vector. When tested in animal model studies, recombinant MVAs have been shown to be avirulent, yet protectively immunogenic as vaccines against viral diseases and cancer (31). Recent studies in macaques severely immuno-suppressed by simian immunodeficiency virus (SIV) infection have further supported the view that MVA should be safe in immuno-compromised humans (35).

MVA is currently in development as a vector for multiple diseases including HIV (36, 37), Tuberculosis (38), Hepatitis C (Barnes et al submitted), influenza (39) and melanoma (40). MVA vectored vaccines are particularly suited to boosting immune responses to an antigen following a priming vaccination with another viral vector (41, 42). In Professor Hill's group, MVA encoding the malaria antigens ME-TRAP (O'Hara et al, submitted. Ewer et al, submitted), CS, polyprotein, merozoite surface protein (MSP)1 (Sheehy et al. submitted) & apical membrane antigen (AMA)1 (Sheehy et al. submitted) has been administered to over 900 individuals, including children and infants in sub Saharan Africa (in more than 190 individuals this followed ChAd63 priming). For adults, the optimal dose and route of MVA ME-TRAP, balancing immunogenicity and reactogenicity, is 2×10^8 pfu intramuscularly.

ChAd63 vector

Adenoviruses are attractive vectors for human vaccination. They possess a genetically stable virion so that inserts of foreign genes are not deleted. Adenoviruses can infect large numbers of

cells without any evidence of insertional mutagenesis. Previous mass vaccination campaigns in very large numbers of US military personnel using orally administered live human adenovirus serotypes 4 and 7 have shown good safety and efficacy (43).

The most widely studied recombinant adenovirus vector is the human adenovirus, AdHu5. However, the ubiquity of human adenovirus infections can generate host anti vector immunity that may limit the utility of this vector. Depending on the geographical region, between 45 – 80 % of adults carry AdHu5-neutralising antibodies (44). Immunisation with AdHu vectors in animal models in the presence of pre-exposure to human adenoviruses attenuates responses to the vaccine (45-47). Phase I trials of a multiclade HIV-1 vaccine delivered by a replication defective AdHu5 have previously excluded volunteers with pre-existing antibodies to AdHu5 at titres greater than 1:12 (48). Higher antibody titres attenuated immunogenicity, although they did not result in higher reactogenicity (49).

The prevalence of immunity to human adenoviruses prompts the consideration of simian adenoviruses as vectors. They exhibit hexon structures homologous to that of human adenoviruses. Simian adenoviruses are not known to cause pathological illness in humans and the prevalence of antibodies to chimpanzee origin adenoviruses is less than 5% in humans residing in the US (50). In a recent study in Kilifi Kenya, 23% of children (aged 1-6 years) had high-titre neutralising antibodies to AdHu5, whilst only 4% had high-titre neutralising antibodies to ChAd63 (51).

There is no available or validated in vitro cell co-culture method to examine co-infection with human and simian adenovirus vectors as the latter are non-replicating. Due to a lack of any sequence homology between the replication-deficient ChAd63 and MVA vectors, complementation of MVA by ChAd63 does not occur. Pre-clinical bioavailability studies have demonstrated no persistence of the ChAd63 vector 24 hours post intramuscular administration. Therefore, residual priming ChAd63 vector is very unlikely to be present at the time of administration of a MVA boost, 8 weeks later.

Clinical studies with ChAd63 ME-TRAP have shown that the intramuscular route of administration is associated with fewer and short lived local adverse events (AEs) and no reduction in immunogenicity, compared with the intradermal route (O'Hara et al submitted).

The ME-TRAP insert

The polypeptide that has been encoded consists of a series of known cytotoxic T-lymphocyte (CTL) epitopes from Plasmodium falciparum pre-erythrocytic stage antigens (52), fused to a complete pre-erythrocytic stage antigen, Thrombospondin Related Adhesion Protein (TRAP) (53). The individual CTL epitopes are recognised by a number of common human leukocyte antigen (HLA) types, represent a variety (six) of potentially protective target antigens and are

included to ensure an immune response to the vaccine in the majority of the population vaccinated (54). TRAP is an abundant pre-erythrocytic stage antigen. Human volunteers immunised with irradiated sporozoites and protected against malaria develop T cell responses against TRAP making it a strong candidate for inclusion in a malaria vaccine (55). Viral vectors containing the CS antigen used in the RTS, S vaccines are much less immunogenic than TRAP, and were not protective in sporozoite challenge studies (56).

Assessing Vaccine Induced Cell Mediated Immunity

When assessing immunogenicity in clinical studies we use the gamma-interferon enzyme-linked immunospot (ELISPOT) assay in two forms. In its ex vivo form this assay correlated directly with protection in two mouse models of malaria (57). In its long-term culture form, it correlated with protection in the field trial of RTS,S/AS02 in the Gambia (58) and in sporozoite challenge studies of viral vector vaccinations in Oxford (59). The ELISPOT enumerates T cells in volunteers' peripheral blood which secrete gamma-interferon on contact with an epitope from the construct. Gamma-interferon secreted by T cells after interaction with infected liver cells has been shown to induce death of liver-stage parasites (60). We will also use the ICS assay to examine the CD8+ IFNγ T cell population that correlated with protection in the recent sporozoite challenge study of ChAd63-MVA ME-TRAP as described above (Ewer et al, submitted) and other cytokine-secreting T cells with different effector functions. Intracellular cytokine staining (ICS) assays allow sensitive, quantitative ex vivo assessments of antigen-specific T cells including immunophenotyping of responding cells and measurement of multiple effector functions.

Rationale

An important next step in the clinical development of ChAd63 / MVA ME-TRAP heterologous prime-boost immunisation is to assess efficacy in the field. The VAC046 clinical trial (SSC Number 2116, Kenya) is planned to commence in 2012 to assess efficacy, immunogenicity and safety of this vaccination strategy in adults in Kenya. The vaccines will be given by the preferred route, intramuscular, and at the preferred adult doses, 5 x 10^{10} vp of ChAd63 ME-TRAP and 2 x 10^{8} vp of MVA ME-TRAP, in the standard prime-boost regimen of ChAd63 ME-TRAP followed eight weeks later by MVA ME-TRAP.

The VAC047 study proposed here will be undertaken in parallel using the same vaccination regimen and overall study design as VAC046, extending the Phase II development of the vaccination strategy to a separate malaria endemic setting. The investigators seek to combine the results of the two studies in a metaanalysis to evaluate the efficacy of the vaccination regimen in a larger sample of adults living in malaria-endemic areas.

There are plans to undertake a clinical trial to evaluate the efficacy of ChAd63 / MVA ME-TRAP prime boost immunization in infants in a malaria endemic area, if justified by the results of these trials. The most efficacious regimen incorporating ChAd63 ME-TRAP and MVA ME-TRAP would be selected for this trial.

6. JUSTIFICATION

This study is justified by the high incidence of malaria mortality and morbidity in Senegal and the rest of sub-Saharan Africa. There is pressing need for an effective vaccination. While insecticide-treated bed nets, vector control measures and new cheap antimalarial drug development are all important aspects of malaria control, a co-existing vaccine development programme is essential. The most advanced malaria vaccine in development (RTS,S) is partially effective, and parallel alternative vaccine development programmes are therefore justified. Furthermore, the virally vectored vaccinations that we will study induce T cell responses to the TRAP antigen: In contrast, RTS,S induces primarily antibody responses to the circumsporozoite antigen. These approaches may be additive, or even synergistic, when used together.

The vaccination strategy proposed here has protected non-immune volunteers in Oxford against heterologous sporozoite challenge, and has the potential to protect against naturally acquired infection in adults in Senegal. If so, the study will provide an accurate estimate of efficacy, and an opportunity to study in detail the correlates of immunity.

7. NULL HYPOTHESIS

There is no delay in time to infection between vaccinees and control vaccinees with *P. falciparum* as determined by PCR based monitoring following curative anti-malarials in malaria-exposed adults in Senegal.

8. OBJECTIVES

Primary objective

To assess the efficacy of a heterologous prime -boost vaccine strategy with ChAd63 ME-TRAP and MVA ME- TRAP in healthy adults in Senegal.

Secondary objectives

To assess the immunogenicity and correlates of efficacy of a heterologous prime -boost vaccine strategy with ChAd63 ME-TRAP and MVA ME-TRAP in healthy adults in Senegal.

To assess the safety and reactogenicity of a heterologous prime -boost vaccine strategy with ChAd63 ME-TRAP and MVA ME-TRAP in healthy adults in Senegal.

To assess the impact of natural immunity (cellular and humoral) on time to re-infection in healthy adults in Senegal

Tertiary objective

To assess the efficacy of a heterologous prime -boost vaccine strategy with ChAd63 ME-TRAP and MVA ME- TRAP in a larger sample of healthy adults living in malaria-endemic regions of Africa by undertaking a metaanalysis combining the results of the proposed study with those of the parallel study, VAC046.

9. STUDY DESIGN AND METHODOLOGY

Study site and population

The town of Guédiawaye is situated on the coastal shoreline in the Dakar region, bordered to the East and the South by the town of Pikine and to the West by the city of Dakar, specifically by the municipal district of Parcelles Assainies. Guédiawaye is situated in the suburbs of Dakar, approximately 10 km from the capital, and covers an area of 30 km² (Figs. 2 and 3). Its population is estimated at 452,168 inhabitants. The average annual rainfall amounts to 600 mm. Over the past few years, malaria transmission has reached record levels in all 5 municipal districts within the *Départment*, namely Médina Gounas, Whakhinane Nimzath, Golf Sud, Sam Notaire and Ndiareme Limamoulaye (Figs. 4 and 5). There are several reasons that account for the resurgence of malaria in the Dakar suburbs:

1. A very high population density.
2. Uncontrolled occupation of low-pressure areas.
3. Lack of a drainage system.
4. Existence of several wetland areas (containment basins, marshland, etc.)
5. Recurrent cyclic flooding since the year 2000.

6. The existence of environments that are particularly conducive to *Anopheles* reproduction.

An investigation carried out by our laboratory revealed an estimated 26 to 32% malarial morbidity, figures that have been confirmed by PNLP (*Programme National de Lutte contre le Paludisme* - National Anti-Malaria Campaign) data. We feel that the primary pre-requisite has been met (intense to moderate malaria transmission).

Fig. 2: Location of Guédiawaye on the Dakar Peninsula

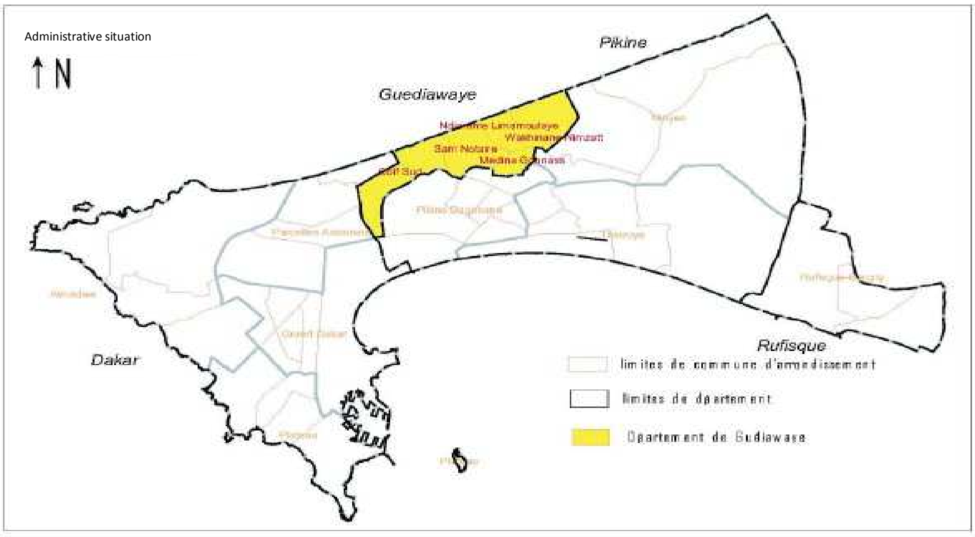

Source: CIEU, 2004.

Fig. 3: Map showing the 5 municipal districts that make up the Guédiawaye *Arrondissement*

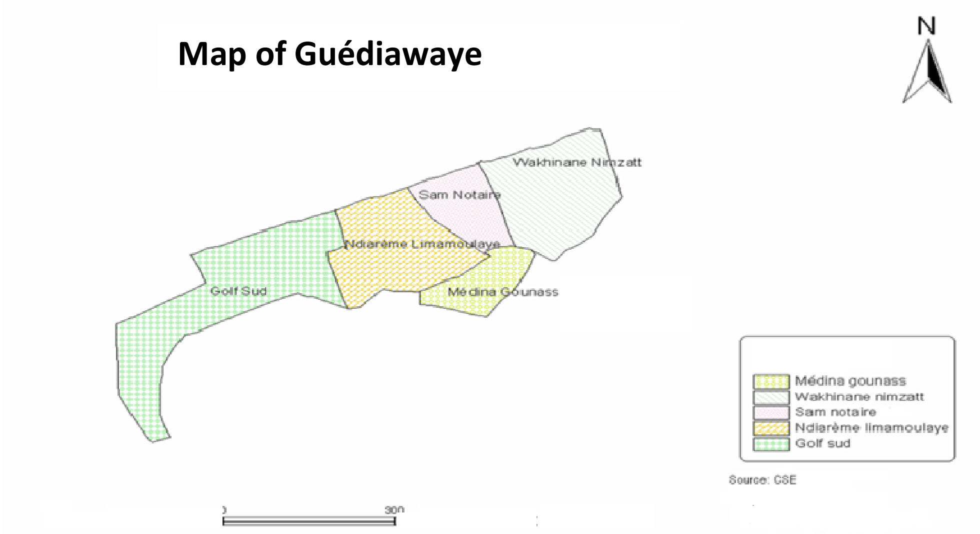

Fig. 4: Position of Guédiawaye on the shoreline, showing low points and vegetation cover

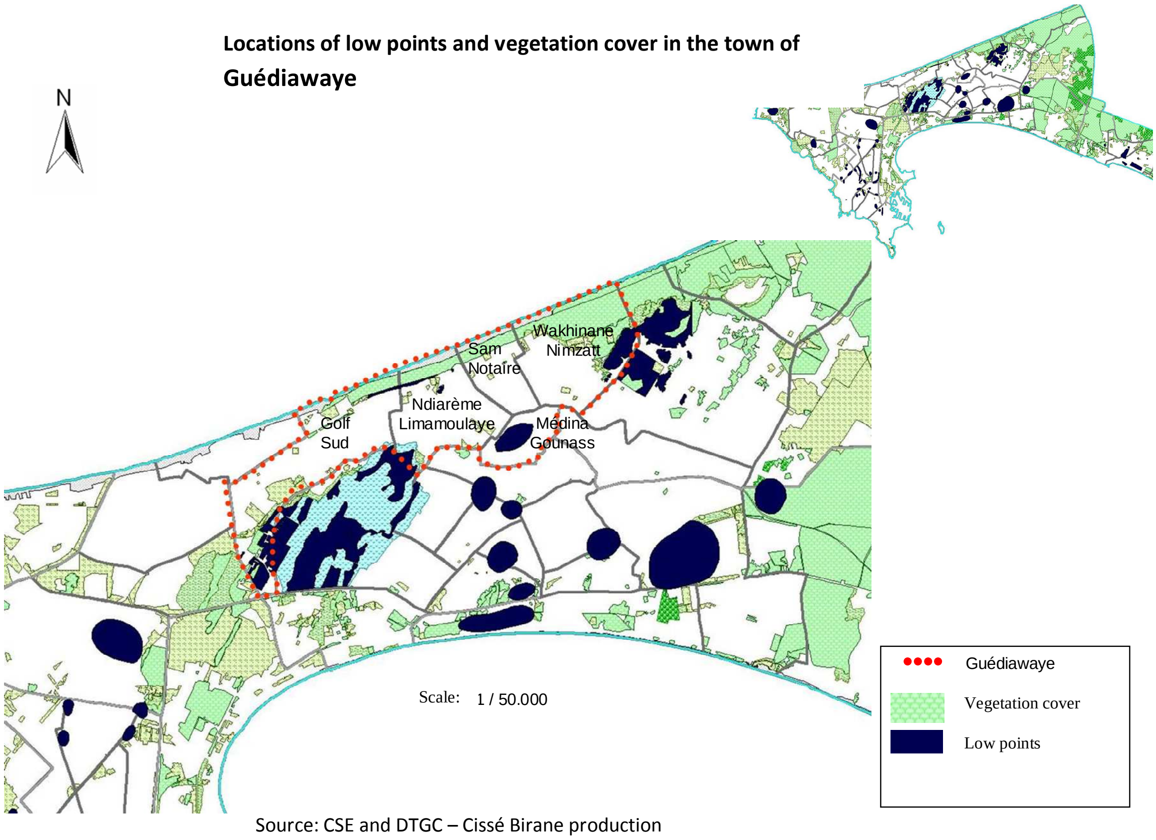

Fig. 5: Pattern of cases of <u>severe malaria</u> in 2005 at the King Baudouin Health Centre, plotted against the pattern of rainfall.

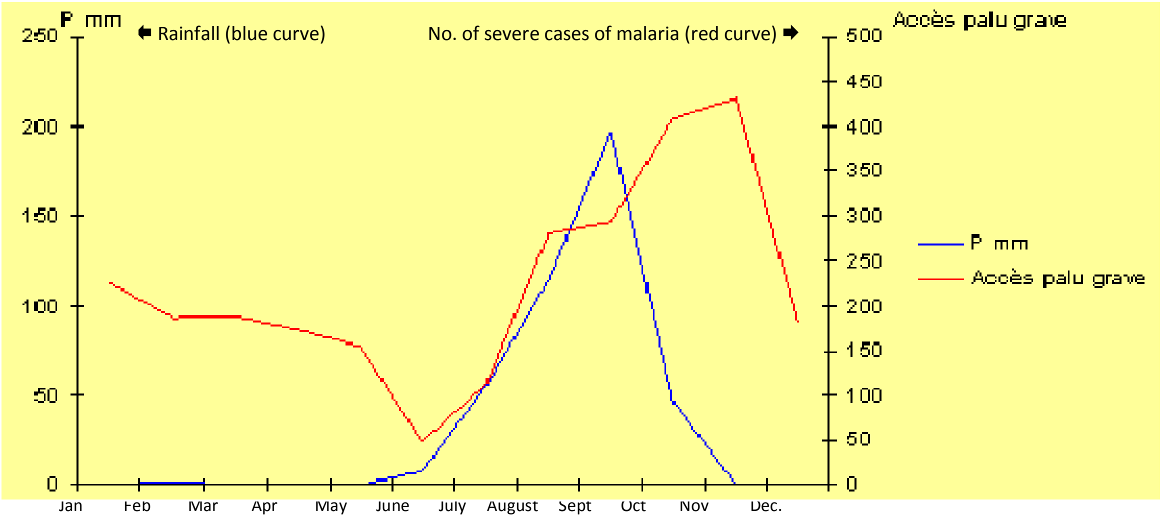

More recent data show a marked tendency to a resurgence of malaria in the area. The number of cases of severe malaria presenting in the region went up from 16 per '000 in 2008 to 19 per '000 in 2009). In the Guédiawaye district, the rate went up from 5 to 12 per '000 (Thesis by Dr Cherif Ba, personal communication)

Methodology

This is a single blinded randomised vaccine trial. The primary focus of the research will be directed at the impact of the proposed vaccine on the numbers of parasite carriers over a 2 month period following vaccination. This period will occur during the 2012 malaria transmission season.

Sensitization and recruitment

The local administrative and community leaders as well as health care workers will be the first to be sensitized about the study. Public meetings will be arranged to inform the communities of the planned study.

During a meeting the investigators will explain the following: the need for a vaccine, the current status of vaccine development, the study screening and informed consent procedure; risks of vaccination and the possible benefits of vaccination. After this meeting field workers will identify potential volunteers, and information sheets translated into relevant local languages will be distributed. The field workers will conduct individual meetings with the participants to ensure understanding of the information sheet and consent form. Individuals who feel that the trial is appropriate for them will be invited to attend a formal screening visit in the study dispensaries. Further discussion will then occur with volunteers in the study dispensaries where the study clinicians will seek informed consent with an experienced field worker.

After consent is obtained, each consented volunteer will be screened for clinically significant acute or chronic disease, using both general physical examination and screening laboratory tests (full blood count (FBC), Hepatitis B, alanine transaminase (ALT) and creatinine. The findings at screening will be used to determine whether or not the volunteer is eligible to participate in the trial. Volunteers eligible to participate in the trial will meet all of the inclusion criteria and none of the exclusion criteria (listed below). Likelihood of migration will be an important factor in determining eligibility to participate in the trial. All volunteers will be informed of results of laboratory tests and referral to an appropriate health facility done where necessary. Volunteers will be informed of the need to conduct anonymous HIV testing. All volunteers will receive pre-test counselling from a trained counsellor who will also do post-test counselling after giving the results. HIV positivity will be an exclusion criterion. In order to maintain the confidentiality of those volunteers infected with HIV, we will make it clear that during screening one can be excluded due to a range of diseases (not just HIV) as well as abnormal laboratory results. Enrolment (receipt of first vaccination) will be done within 90 days of screening. Volunteers enrolled will be given photo identity (ID) cards for ease of identification and also to serve as a reminder of the appointment dates. These will be destroyed at the end of the study.

Vaccination, observation and blood sampling will occur at King Baudouin hospital.

Study groups

Subjects will be randomized to receive either ChAd63 ME-TRAP and MVA ME-TRAP or control vaccination with Rabies vaccine. To preserve blinding, Rabies vaccine will be administered in a 0, 2 month schedule. This differs from the standard schedule for rabies vaccination, however, administration of rabies vaccine by alternative schedules may still provide some protective efficacy against rabies[63]. In addition, all participants will be offered a standard schedule of Rabies vaccine after the trial comes to an end.

Simple randomisation into the study groups will be done by an independent statistician and the study clinicians with an allocation ratio of 1:1 to study groups taking into account their area of residence. A randomization code list will be generated by an independent statistician and its use guided by a clear standard operating procedure (SOP). Allocation concealment will be employed by use of opaque sealed envelopes. The volunteers, fieldworkers and laboratory scientists will be blinded to vaccine allocation until the end of the study, but the clinical investigators will not be blinded.

All vaccinations will be administered by the intramuscular route – IM. Randomization into groups will be as follows:

- **Group 1**(60 volunteers): ChAd63 ME-TRAP 5 $\times 10^{10}$ viral particles (vp) followed by MVA ME–TRAP 2 $\times 10^{8}$ plaque forming units (pfu) 8 weeks later.
- **Group 2**(60 volunteers): control, i.e. Rabies vaccine1ml followed by Rabies vaccine 1ml 8 weeks later.

Oral antimalarials will be taken by all participants from days 63-65 inclusive. The purpose is to clear any asymptomatic parasitaemia (both asexual blood stage parasites and gametocytes) prior to the commencement of PCR monitoring for new malaria infection from Day 70. A standard 3 day course of Atovaquone/proguanil will be combined with three days of concurrent oral artesunate. Atovaquone/proguanil has been chosen over artemether/lumefantrine to limit the potential for persistence of antimalarial drug from Day 70 when monitoring for new malaria infection commences (lumefantrine has a relatively long elimination half life[65]). Artesunate has been selected because of its antigametocidal activity. The anti- malarial treatment will be directly observed.

The control group will undergo similar tests to the vaccinated groups (such as ex-vivo and cultured ELISPOT and ICS) to control for T cell responses to TRAP and other antigens which may develop during the study period as a result of natural exposure to *P. falciparum.*

All volunteers will follow the same schedule for clinic visits and field worker visits, vaccinations (Rabies vaccine or ChAd63 ME-TRAP / MVA ME-TRAP, depending on group), antimalarial treatment, and intensive PCR monitoring. There will be a total of 24 clinic attendances, 22 blood

sampling occasions (including finger-prick samples), 2 field workers home visits and two vaccination visits for each volunteer.

Selection and withdrawal of subjects

We will continue to recruit and screen volunteers until at least 120 eligible volunteers have been identified, preferably until 130 eligible volunteers have been identified. If volunteers withdraw consent prior to receiving their first vaccination, we will screen and recruit in order to replace the number withdrawing consent.

Inclusion Criteria

- Consenting adult males aged 18 – 50 years in good health.
- Will remain resident in the study area for the study duration.
- Able and willing (in the Investigator's opinion) to comply with all study requirements
- Informed Consent

Exclusion criteria

Any of the following constitutes exclusion criterion:

- Any significant medical disease, disorder or finding which may significantly increase the risk to the volunteer because of participation in the study, affect the ability of the volunteer to participate in the study or impair interpretation of the study data
- Hypersensitivity to Rabies vaccine,the trial vaccines or the antimalarial used.
- History of allergic disease or reactions likely to be exacerbated by any component of the vaccines, e.g. egg products, kathon, neomycin
- History of splenectomy.
- Haemoglobin less than 10.0 g/dl
- Clinically significant abnormalities of laboratory screening tests (full blood count, ALT, creatinine levels).
- Blood transfusion within the month preceding enrolment.
- History of vaccination with previous experimental malaria vaccines or other vaccines likely to impact on findings of study (e.g. other MVA or adenovirus vectored vaccines)
- Administration of any other vaccine or immunoglobulin within 2 weeks before vaccination.
- HIV or Hepatitis B surface antigen seropositivity.

- Current participation in another clinical trial or recent participation within 12 weeks of this study.
- Any other finding which in the opinion of the investigators would increase the risk of an adverse outcome from participation in the trial.
- Likelihood of travel away from the study area

Withdrawal criteria

Subjects may be withdrawn from the study:

- By withdrawing consent
- On the decision of the investigator
- On the advice of the data safety and monitoring board (DSMB)

The investigator may withdraw the subject for the following reasons:

- Any adverse event which results in the inability to comply with study procedures
- Ineligibility either arising during the study or retrospectively (having been overlooked during screening)
- Significant protocol deviation.
- Loss to follow up (applies to a subject who consistently does not return for protocol study visits, is not reachable by telephone or any other means of communication and/ is not able to be located).

The reason for withdrawal will be recorded in the case report form (CRF).

If a subject is withdrawn for any reason, the reason will be recorded. If withdrawal is the result of a serious AE, the investigator will offer to arrange for appropriate specialist management of the problem and the ethical committee will be informed in a timely manner. The extent of follow up will be determined by a medically qualified investigator, but will be at least for the whole study period. Subjects withdrawn prematurely for any reason will not receive further vaccinations, although they may be requested to come back to the clinic for safety evaluation.

Study schedule and procedures

Screening visit

Volunteers will be provided with detailed information about the study, as described in the attached information sheet. Consent will be written. After informed consent has been obtained the study clinicians will take a clinical history, examine all volunteers carefully and conduct a number of standard laboratory tests (FBC, ALT, rapid HIV tests, creatinine and Hepatitis B surface antigen test) to screen subjects for clinically significant acute or chronic illnesses. Those with abnormal results will be offered appropriate investigations and treatment or referral as

necessary. A photograph will also be taken in order to produce a study identification card which will be given to the volunteer. The electronic stored version of the photograph will be identified by the unique study number only and destroyed at the end of the study.

HIV testing

Volunteers will have access to a trained counsellor who will do pre and post test counselling. HIV sero-status will be established using the standard rapid diagnostic kits and testing algorithm used by the Senegalese Ministry of Health and Prevention. Those diagnosed positive will be referred to the HIV Department of King Baudouin hospital for further counselling and treatment.

Enrolment

Volunteers are considered enrolled into the study when they receive their first vaccination. This should occur ≤ 90 days after the screening visit. They will remain in the vaccinating health facility for 30 minutes post vaccination in order to enable monitoring by the study clinicians. The vaccination health facility will have resuscitation equipment and drugs available during vaccination clinics. The study clinicians will assess and record local as well as systemic adverse events after 30 minutes have elapsed.

Follow- up

Participants will be followed up for approximately 6 months after the first vaccination. Study clinicians and field workers supervised by medical officers will assess and record local adverse events post vaccination including pain, swelling and discolouration. He or she will then assess and record systemic adverse events including temperature, malaise and nausea. Each side effect will be classified as absent, mild, moderate or severe. Each volunteer will be seen 2 weeks after ChAd63 ME-TRAP vaccination and 1 week after MVA ME-TRAP vaccination for a full safety and reactogenicity assessment by a study clinician. An additional visit at home will be made by a field worker (reporting to the study clinicians) on day 1 after each vaccination, and if necessary daily as directed by a study clinician. The study clinicians will review reports from home visits conducted by field workers, and arrange to see the volunteers in person if appropriate.

The volunteers will be asked to present themselves to the study Hospital if they develop any illnesses during follow up. Any emerging safety data that is considered cause for concern by the DSMB will be relayed to the participants during the study visits.

Table 1: Solicited local and general adverse events to be documented in the CRF

**Adverse events**	
**Local (injection site)**	Pain at the injection site
	Redness at the injection site
	Swelling at injection site
	Warmth at the injection site
**General**	Documented fever (axillary temperature > 37.5° C)
	Symptoms of feverishness
	Malaise
	Arthralgia
	Headache
	Myalgia
	Nausea / vomiting
	Other(specify)

Blood sampling

More detailed immunological studies (details below) will be conducted on a nested cohort, which will require larger volume blood draws at certain time points (details below). In order to identify this nested cohort, participants will be further divided into 2 sampling groups: Group A (30 participants) and B (90 participants). Allocation to sampling groups (i.e. A vs. B) shall be done randomly and stratified by vaccination group (i.e. 1 ChAd63/MVA ME-TRAP vaccinee for each control vaccinee).See tables 2 and 3 below.

20 mls of blood will be collected at screening (similar for both sampling groups) to test eligibility, which will be used for;

- Full blood count
- Serum ALT and creatinine
- HIV antibody testing (pre vaccination only) and Hepatitis B surface antigen
- Baseline *P. falciparum* PCR assay

40 mls of blood will be collected on days 0, 14, 63 and 161 for group A and at day 63 for group B. The following tests will be performed at these time points;

- Full blood count
- Serum ALT and creatinine

- Cellular immunology studies
- Plasma for antibody studies of immunity to malaria antigens.

10 mls of blood will be collected at day 56 for group A and at days 0, 14, 56, and 161 for group B. The following tests will be performed at these time points;

- Full blood count
- Serum ALT and creatinine

10mls of blood will be collected at Days 1 and 57 for Group A and stored for possible future gene expression studies.

Blood samples will be collected at day 63 and then between day 70 and day 119 for PCR monitoring. From day 70, blood samples will be collected three times per week for 4 weeks and then once per week for a further 4 weeks. Each blood sample will be taken by fingerprick (volume, 0.5-1.0ml), or, if the volunteer prefers, by venepuncture (volume, 2ml). See table 4 below for details of timing.

Where possible, blood samples will be stored for later immunological studies. This will allow further characterisation of the set of lymphocytes responding to the vaccine, and for exploratory immunology assessments to be done at the discretion of the investigators. Exploratory immunology assessments may include the following: HLA typing; DNA analysis of genetic polymorphisms potentially relevant to vaccine immunogenicity using sequencing or other genotypic methods; gene expression profiling; B cell ELISPOT; cytokine analysis; antivector immune responses; ELISA for measurement of antibodies to TRAP; and in vitro growth inhibition assays.

Table 2: Group A sampling volumes (30 participants)

**Clinic visit**	**1**	**2**		**3**	**4**		**7**	**8-19**	**20-23**	**24**
**Field Worker Visit**			**1**			**2**				
**Timeline (days)***	S	D0	D1	D14	D56	D57	D 63	Week 11: D70, 72, 74<br>Week 12: D77, 79, 81<br>Week 13: D84, 86, 88<br>Week 14 : D91, 93, 95	Week 15: D98<br>Week 16: D 105<br>Week 17: D112<br>Week 18: D119	D161
**Blood volume (mL)**	20	40	10	40	10	10	40	(0.5 – 2,0) x 12 = 6 – 24	(0.5 – 2.0) x 4 = 2 – 8	40
**Cumulative blood volume (mL)**	20	60	70	110	150	160	200	206-224	208-232	248-272

Table 3: Group B sampling volumes (90 participants)

Clinic visit	1	2	3	4	7	8-19	2-23	24
**Timeline (days)***	S	D0	D 14	D56	D63	Week 11: D70, 72, 74<br>Week 12: D77, 79, 81<br>Week 13: D84, 86, 88<br>Week 14 : D91, 93, 95	Week 15: D98<br>Week 16: D 105<br>Week 17: D112<br>Week 18: D119	D161
**Blood volume (mL)**	20	10	10	10	40	(0.5 – 2.0) x 12 = 6 - 24	(0.5 – 2.0) x 4 = 2 - 8	10
**Cumulative blood volume (mL)**	20	30	40	50	90	96-114	98-122	108-132

<u>Laboratory procedures</u>

Because the laboratory immunology testing is expensive and labour intensive, cellular immunology will be conducted on all participants at the critical pre-surveillance time point only (i.e. day 63). We will randomly select 30 individuals (stratified by vaccination group) for cellular immunology studies on days 0, 14, and 161.

Plasma and cells will be stored at -20°C and -192°C respectively. ELISPOTs and fluorescence - activated cell sorter (FACS) studies following intracellular cytokine staining will be performed, and results validated by rigorous use of negative and positive controls. This technique uses an overnight stimulation by antigen of separated lymphocytes from the volunteer's blood sample to count the number of interferon gamma producing cells. Analysis by FACS, where sufficient numbers of lymphocytes are isolated, will allow further characterisation of the response, by co-staining lymphocytes with CD4 and CD8 as well as IFN gamma and other cytokines.

PCR studies will be undertaken firstly at King Baudouin hospital of Guediawaye. Blood will be filtered to remove white blood cells, and then DNA extracted from the full volume of blood. Real time PCR will be undertaken at UCAD, and a sub-set of the samples will be stored for further quality control studies in Oxford. This is because the method to be used at UCAD has been validated in Oxford.

Vaccine handling and administration

Final batch certification and associated labelling of the IMPs, ChAd63 ME-TRAP and MVA ME-TRAP, is performed by the Qualified Person at the Clinical Biomanufacturing Facility (CBF), Churchill Hospital, Oxford, in accordance with Annex 13 and the CBFs internal SOPs.

Accountability, storage, shipment and handling of IMPs will be in accordance with relevant SOPs and forms. All movements of IMPs will be documented in vaccine accountability logs according to local site SOPs.

The IMPs are stored between $–70^0$C and $–90^0$C in a locked freezer at the University of Oxford, Churchill Hospital. The vaccines will be shipped from Oxford on dry ice then stored at $–70^0$C in a locked freezer at UCAD until required. Vaccines will be thawed on the morning of use, and kept in a cold box in the field. They will be used within 4 hours of thawing.

The rabies vaccine will be sourced locally. The rabies vaccines will be stored and used in accordance with the manufacturer's instructions.

All ChAd63 ME-TRAP, MVA ME-TRAP and rabies vaccinations will be given by intra-muscular injection. A 23 gauge (G) needle and 1 ml syringe will be used.

Each volunteer will be monitored for 30 minutes (or longer if necessary) after each vaccination. Resuscitation (including intubation) equipment and medication will be available in the clinic site and a clinician trained in resuscitation present at all times.

Indications for delayed vaccination.

The following adverse events constitute contraindications to administration of vaccine at that point in time; if any one of these adverse events occurs at the time scheduled for vaccination, the subject may be vaccinated at a later date, or withdrawn at the discretion of the investigator. The subject must be followed until resolution or stabilization of the adverse event or until causality is determined to be unrelated to trial interventions, as with any adverse event.

- Acute disease at the time of vaccination. (Acute disease is defined as the presence of moderate or severe illness with or without fever). Vaccines can be administered to persons with a minor illness such as diarrhoea, mild upper respiratory infection with or without low-grade febrile illness, i.e., temperature of <37.5°C/99.5°F).
- Temperature of ≥37.5°C (99.5°F) at the time of vaccination.

NB: Anaphylactic reaction following administration of study vaccine constitutes an absolute contraindication to further administration of vaccine, and the subject must be withdrawn and followed until resolution of the event

Table 4: Summary of study visits

Clinic visit	1	2		3	4		5	6	7	8-19	20-23	24
**Field Worker Visit**			**1**			**2**						
**Timeline (days)***	S	D0	D1	D14	D56	D57	D63	D64	D65	Week11: D70,D72,D74<br>Week 12: D77,D79,D81<br>Week13: D84 ,D86,D88<br>Week14: D91, D93,D95	Week 15: D98<br>Week 16: D105<br>Week 17: D112<br>Week 18: D119	D161
**Window period (days)***	-90 to -1			±7	±7		-1 / +7			-1/+4	-2/+4	-7/+7
**Vaccination**		1			2							
**Inclusion/ exclusion criteria**	×											
**Informed consent**	×											
**Medical history**	×											
**Physical exam**	×											
**Safety Bloods^**	×	×		×	×		×					×
**Contraindications review**	×	×			×							
**Local & systemic events review**		×	x	×	×	x	×			×	×	×
Treatment with anti-malarial							×	×	×			
***P.falciparum* PCR**		×					×			×	×	
Immunogenicity Tests #		×		×			×					×
**Blood sample for gene expression @**			x			x						

^The following lab tests will be done: FBC, ALT, Creatinine.

Hepatitis B surface antigen test & HIV testing will also be done at screening.

*****Timeline is approximate only. Exact timings of visits relate to the previous visit – *i.e.* each visit must occur the specified number of days after the last visit ± time window
*All scheduled clinic visits will be done on week days
Immunogenicity tests: 30 volunteers at D0, 14 and 161; all participants at D 63
@ Blood sample for gene expression: 30 volunteers at D1, and 57. This will be as close to 24 hours post vaccination as practicable.

<u>Study Visits</u>

Screening visit: (Clinic visit)

Medical history and physical examination will be performed at screening to exclude any significant medical conditions. The following lab tests will be done: FBC, ALT, rapid Hepatitis B surface antigen test, Creatinine and HIV testing.

D0 (Vaccination with ChAd63 ME–TRAP or Rabies vaccine) (Clinic visit)

Medical history, temperature monitoring +/- physical examination will be performed and eligibility reviewed by the study clinicians prior to vaccination. Venepuncture for exploratory immunology (for 30 volunteers), safety assessment (FBC, ALT and Creatinine- all participants) and baseline PCR for *P. falciparum* for all participants will also be done prior to vaccination.

D 1 (Field worker visit)

Each volunteer will be visited at home on day 1 by a field worker for assessment and recording of any solicited and unsolicited AEs in diary cards. If necessary, the volunteers will continue to be seen regularly until the symptom(s) have resolved or stabilised.

On day 1 the use of an insecticide treated net (ITN) by the volunteer will be assessed by observation in the homestead. Venepuncture for gene expression studies will be done on the 30 Group A volunteers, as close to 24 hours post-vaccination as practicable.

D14 (-/+7 days) (Clinic visit)

Medical history, temperature monitoring +/- physical examination will be performed and recorded. Any solicited/ unsolicited AEs will be recorded. Venepuncture will be performed for FBC, ALT, Creatinine (all participants) and exploratory immunology (for 30 volunteers).

D56 (Vaccination with MVA ME–TRAP or Rabies vaccine) (-/+7 days) (Clinic Visit)

Medical history, temperature monitoring +/- physical examination will be performed and recorded. Venepuncture will be performed for FBC, ALT and Creatinine (all participants). Eligibility will be reviewed by the study clinicians prior to vaccination.

D 57 (Field worker visit)

Each volunteer will be visited at home on day 57 by a field worker for assessment and recording of any solicited and unsolicited AEs in the diary card. If necessary the volunteer will continue to be seen regularly until the symptom(s) have resolved or stabilised. Venepuncture for gene expression studies will be done on the 30 Group A volunteers, as close to 24 hours post-vaccination as practicable.

D63 (-1/+7 day) (Clinic Visit)

Medical history, temperature monitoring +/- physical examination will be performed and recorded. Any solicited and unsolicited AEs will be recorded. Venepuncture will be performed for FBC, ALT, Creatinine, *P.falciparum* PCR analysis and exploratory immunology (for all participants).

1st day of Atovaquone/proguanil + Artesunate therapy

D64 and 65 (Clinic Visit)2nd and 3rd day of Atovaquone/proguanil + Artesunate therapy

Week 11, 12, 13 and 14

Medical history, temperature monitoring +/- physical examination will be performed and recorded. Any solicited and unsolicited AEs will be recorded. Blood samples will be taken by finger-prick or venepuncture for *P.falciparum* PCR analysis.

Week 15, 16, 17 and 18

Medical history, temperature monitoring +/- physical examination will be performed and recorded. Any solicited and unsolicited AEs will be recorded. Blood samples will be taken by finger-prick or venepuncture for *P.falciparum* PCR analysis.

Day 161 (±7 days) (Clinic Visit)

Medical history, temperature monitoring +/- physical examination will be performed and recorded. Any solicited and unsolicited AEs will be recorded. Venepuncture will be

performed for FBC, ALT, Creatinine (all participants) and exploratory immunology (for 30 participants).

Concomitant therapy

Any concomitant medication taken during the study period will be recorded.

Endpoints

Primary Endpoint – Vaccine Efficacy

We will compare active and control vaccination for time to first episode of *P.falciparum* infection, defined as 2 or more consecutive blood samples confirmed positive by PCR, for *P.falciparum*.

Secondary Endpoints

Measures of immunogenicity will include:

Ex vivo ELISPOT responses to overlapping pools of ME – TRAP peptides.

Cultured ELISPOT responses to overlapping pools of ME – TRAP peptides.

ICS by flow cytometry for cell mediated immune responses

ELISA for antibodies to malaria antigens

All solicited and unsolicited local and systemic vaccine- linked adverse events (AEs) including clinically significant laboratory abnormalities.

Tertiary Endpoint

Metaanalysis of Vaccine Efficacy: We will compare combined active vaccination from VAC046 and VAC047, with combined control vaccination from VAC046 and VAC047, for time to first episode of *P.falciparum* infection, defined as 2 or more consecutive blood samples confirmed positive by PCR for *P.falciparum*.

10. SAFETY

Safety oversight will be the responsibility of the investigators and the independent Data Safety and Monitoring Board (DSMB).

Discontinuation of the study

Both arms of the study will be discontinued in the event of the following:

- New scientific information is published to indicate that the volunteers in the study are being exposed to undue risks as a result of administration of investigational medicinal products (IMPs) or as a result of the follow –up schedule.
- Serious concern about the safety of the IMPs arise as a result of vaccine related serious adverse event(SAE)s occurring in the subjects enrolled in this or any other ongoing study of the IMPs.
- For any other reason at the discretion of the Principal investigators.

Data collection

Adverse events will be documented in individual case report forms (CRFs) for each volunteer. They will be recorded under two headings; local and general. There will be separate sections for the concomitant medication, concomitant vaccinations, non–serious adverse event documentation, serious adverse event documentation and study conclusion. Any deviations from the study protocol will be documented. Case report forms will be kept securely, and HIV status will not be recorded in the CRF.

Definitions of adverse event categories

Adverse event

Any untoward medical occurrence in a patient or clinical investigation subject occurring in any phase of the clinical study whether or not considered related to the vaccine. This includes an exacerbation of pre-existing conditions or events, intercurrent illnesses, or vaccine or drug interaction. Anticipated day-to-day fluctuations of pre-existing conditions, including the disease under study, that do not represent a clinically significant exacerbation will not be considered adverse events. Discrete episodes of chronic conditions occurring during a study period will be reported as adverse events in order to assess changes in frequency or severity.

Adverse events will be documented in terms of a medical diagnosis(es). When this is not possible, the adverse event will be documented in terms of signs and symptoms observed by the investigator or reported by the subject at each study visit.

Pre-existing conditions or signs and/or symptoms (including any which are not recognised at study entry but are recognised during the study period) present in a subject prior to the start of the study will be recorded on the Medical History form.

Adverse reactions

In the pre-approval clinical experience with a new medicinal product or its new usages, particularly as the therapeutic dose(s) may not be established, all noxious and unintended responses to a medicinal product related to any dose should be considered adverse reactions.

Serious Adverse Events (SAE)

A serious adverse event is any untoward medical occurrence that at any dose:

- results in death,
- is life-threatening,

  Note: The term "life-threatening" in the definition of "serious" refers to an event in which the patient was at risk of death at the time of the event; it does not refer to an event which hypothetically might have caused death if it were more severe.
- requires inpatient hospitalisation or prolongation of existing hospitalisation,
- results in persistent or significant disability/incapacity, or
- is a congenital anomaly/birth defect.

Medical and scientific judgment should be exercised in deciding whether expedited reporting is appropriate in other situations, such as important medical events that may not be immediately life-threatening or result in death or hospitalisation but may jeopardise the patient or may require intervention to prevent one of the other outcomes listed in the definition above. These should also usually be considered serious.

SAEs are subject to expedited reporting to the sponsor, ethics committee and local safety monitor (see below).

Suspected Unexpected Serious Adverse Reactions (SUSAR)

An adverse reaction, the nature or severity of which is not anticipated based on the applicable product information (*e.g.*, Investigator's Brochure for an unapproved investigational medicinal product) is considered as an unexpected adverse drug reaction. Where the adverse reaction is also considered to have a possible, probable or definite relationship with the vaccine, and also meets the criteria for a serious adverse reaction, it is termed a Suspected Unexpected Serious Adverse Reaction (SUSAR). These events are subject to expedited reporting as for SAEs, and are also reported to the regulatory authority (see below).

<u>Collection of Adverse events</u>

At each post-vaccination visit an examination will be documented including an examination of local reactions at the injection site. The largest diameter through the injection site of any redness will be recorded in millimetres. The largest diameter through the injection site of local swelling, defined as a more generalized swelling of the deltoid muscle will be recorded in millimetres. Severity of these local findings will be graded using the scale given in Table 4.

Table 4: Grading a) for swelling

Grade	**Diameter [mm]**
0	0
1	< 20
2	20 – 50
3	> 50

b) for redness

Grade	**Diameter [mm]**
0	0
1	< 50
2	50 – 100
3	> 100

Study subjects will be asked to indicate the maximum degree of pain they experience at the injection site using a scale ranging from 0 to 3 as described in Table 5.

Table 5: Pain scale

**Grade**	**Description**
**0**	No pain at all
**1**	Painful on touch, no restriction in movement of arms, able to work, drive, carry heavy objects as normal
**2**	Painful when limb is moved<br>(*i.e.* restriction in range of movement in arm, difficulty in carrying objects)
**3**	Severe pain at rest<br>(*i.e.* unable to use arm due to pain.)

All local reactions will be considered causally related to the vaccination in the absence of another more likely explanation (such as recent trauma).

At each visit subjects will be requested to report local and general side effects they might have experienced since they last were seen. The investigator will assess the severity of the solicited signs and symptoms using the key provided in Table 6.

Table 6: Intensity of the general adverse events will be assessed as described:

GRADE 0	None
GRADE 1	Mild: Transient or mild discomfort (< 48 hours); no medical intervention/therapy required
GRADE 2	Moderate: Mild to moderate limitation in activity - some assistance may be

	needed; no or minimal medical intervention/therapy required
GRADE 3	Severe: Marked limitation in activity, some assistance usually required; medical intervention/therapy required, hospitalisation possible

The investigator will use the guidelines provided below to assess the relationship of the event to the administration of the vaccine. Both severity of the event and its relationship to the vaccine administration will be documented in the CRF.

Further details for any AE (such as start/stop date and any treatment), will be gathered, regardless of the relationship to the vaccine. We will also document any unsolicited adverse event reported by the volunteer. Serious adverse events (SAE) as defined above will be documented and reported using a serious adverse event reporting form.

Follow-up of Adverse Events

Adverse events likely to be related to the vaccine, whether serious or not, which persist at the end of the trial will be followed up by the investigator until their resolution or stabilisation or until causality is determined to be unrelated to trial interventions.
Moreover, any serious adverse event likely to be related to the vaccine and occurring after trial termination should be reported by the investigator according to the procedure described below.
Outcome of any non-serious adverse event occurring within 30 days post-vaccination (*i.e.* unsolicited adverse event) or any SAE reported during the entire study will be assessed as:

- Recovered/resolved
- Not recovered/not resolved
- Recovering/resolving
- Recovered with sequelae/resolved with sequelae
- Fatal (SAEs only)

Subjects who have moderate or severe on-going adverse events that are not vaccine linked will be referred to an appropriate hospital/health facility at the completion of the study and will be advised to consult a primary care physician if the event is not considered to be related to the study vaccine. A follow-up visit will be arranged to manage the problem and to determine the severity and duration of the event, if it is considered to be related to the study vaccine. If appropriate, specialist review within the King Baudouin hospital of Guediawaye will be arranged.

Reporting of Adverse Events

Every SAE occurring throughout the trial must be reported by telephone, e-mail or fax to the sponsor and DSMB by the investigator as soon as (s)he is alerted of it and within one working

day, even if the investigator considers the AE not related to vaccination. The investigator will then complete a SAE report as soon as possible and within 5 working days or 7 calendar days.

Any relevant information concerning the adverse event that becomes available after the SAE report form has been sent (outcome, precise description of medical history, results of the investigation, copy of hospitalisation report, etc.) will be forwarded to the sponsor in a timely manner, the anonymity of the subjects shall be respected when forwarding this information. Any study-related SUSAR or serious adverse event related to participation in the study must be reported to the ethical review committee (ERC) via email (bathie65@yahoo.fr) within twenty four (24) hours after the principal investigator (PI) becomes aware of the event. The hard copies of the report must be forwarded to the ERC Secretariat within three (3) working days of the initial notification. Follow-up reports should be submitted as soon as more information becomes available. Periodic safety reports of any study-related expected adverse event or adverse events not related to participation in the study will be forwarded to ERC.

Initial reports of SUSARs should be provided by the Sponsor to the Direction de la Pharmacie et des Laboratoires (DPL) as soon as possible but within seven calendar days of the notification of the SUSARs with follow up reports being provided within a further eight (8) calendar days.

The sponsor pledges to inform the authorities of any trial discontinuation and specify the reason for discontinuation.

The causal relationship between the SAE and the product will first be evaluated by the investigator with the following scale (table 7):

Table 7: *Guidelines for assessing the relationship of vaccine administration to an AE*

0	**No Relationship**	No temporal relationship to study product ***and***<br><br>Alternate aetiology (clinical state, environmental or other interventions); ***and***<br><br>Does not follow known pattern of response to study product
1	**Unlikely**	Unlikely temporal relationship to study product ***and***<br><br>Alternate aetiology likely (clinical state, environmental or other interventions) ***and***<br><br>Does not follow known typical or plausible pattern of response to study product.
2	**Possible**	Reasonable temporal relationship to study product; ***or***<br><br>Event not readily produced by clinical state, environmental or other interventions; ***or***<br><br>Similar pattern of response to that seen with other vaccines

3	**Probable**	Reasonable temporal relationship to study product; ***and***<br><br>Event not readily produced by clinical state, environment, or other interventions ***or***<br><br>Known pattern of response seen with other vaccines
4	**Definite**	Reasonable temporal relationship to study product; ***and***<br><br>Event not readily produced by clinical state, environment, or other interventions; ***and***<br><br>Known pattern of response seen with other vaccines

Dissemination and explanation of blood results

All non-immunological blood results will be given to all participants after screening and at follow-up visits. Immunological results will be explained in general terms, for the group not for individuals, at the end of the study. Those with clinically significant abnormal blood results at screening will be offered appropriate investigations and treatment or referral as necessary.

11. TRIAL GOVERNANCE

Oxford University will sponsor the study, and provide insurance for the trial. Regular monitoring will be performed according to ICH Good Clinical Practise and a Monitoring Plan. The monitors will check whether the clinical trial is conducted and data are generated, documented and reported in compliance with the protocol, good clinical practice (GCP) and the applicable regulatory requirements.

The sponsor will convene a Data Safety Monitoring Board that will review the safety data. A local safety monitor will be recruited by the investigators in UCAD, whose role will be to communicate with the DSMB and provide independent assessments of unusual or serious adverse events should it be requested by the DSMB or by the investigators. SAEs will be reported (by the investigators) to the local safety monitor, sponsor and to the Senegalese EC within one working day of the investigators becoming aware of them. The Principal Investigator will be responsible for reporting of SAEs to the DSMB in regular updates. The investigators will be responsible for reporting a summary of safety data at the end of the trial to the Senegalese Direction de la Pharmacie et des Laboratoires, and will report SUSARs and SAEs deemed causally related to the study vaccine to the DPL as stated above.

The DSMB will be empowered to request vaccinations be halted or discontinued, and will report to the sponsor. The investigators will inform the Ministry of Health and Prevention EC and the Pharmacy and Poisons Board if the trial is discontinued, with a full explanation.

The members of the Data Safety Monitoring Board (DSMB) are as follows:

a) Chair: Professor Geoffrey Targett: Emeritus Professor of Immunology of Parasitic Diseases at the London School of Hygiene & Tropical Medicine, Room 410, Keppel Street, London WC1E 7HT, UK

b) Dr Brian Faragher, Liverpool School of Tropical Medicine, UK

c) Professor Mahamadou Thera: MRTC, Bamako, University of Mali, Colline de Badalabougou, BP E2528, Mali

d) Dr Bernhards Ogutu, KEMRI, P.O. Box 2254 – 00202, Nairobi, Kenya

The internal monitor is Dr Allé Baba Dieng, consultant, Sicap liberté 4 n° 5044, telephone +221: 779183195, email: allebabad@yahoo.fr

The external monitor (currently visiting UCAD) is Dr Ceri McKenna, Consultant, Appledown, Oxford, Office tel. +4401494 867676 Mobile 07941 271635, Orchard End, Greenlands Lane Prestwood Gt Missenden, Bucks, HP16 9QX, United Kingdom, email: ceri.mckenna@appledown.net

12. DATA HANDLING AND RECORD KEEPING

Data management

Data management will be conducted in Ucad, and a copy of the anonymized database provided to the sponsor for archiving. The PI will be responsible for receiving, entering, cleaning, querying, analysing and storing all data that accrues from the study. Responsibility for this may be delegated to the study data manager. The data will be entered into the subjects' source documents or offline database. Data will be subsequently transferred to an electronic database for analysis.

If any changes to the study are necessary during the study a formal amendment will be presented to the sponsor prior to submission to the relevant ethical and regulatory agencies for approval unless to eliminate an immediate hazard(s) to study participant without prior ethics approval. Any unforeseen and unavoidable deviations from the protocol will be documented and filed in as a protocol deviation in the Trial Master File, with explanation.

Data capture methods

Data will be captured on to paper source documents for the scheduled clinic visits and home visits, the screening visits, unscheduled clinic visits, and lab and other investigational results.

The paper source documents will be kept securely, but may be taken to the field for direct data entry. Data entry from paper records onto a database will be conducted at UCAD. Immunological data will be transferred to an electronic database for analysis without any volunteer identifier apart from the unique volunteer number.

Archiving

The investigator must keep all trial documents for at least 15 years after the completion or discontinuation of the trial.

Protocol deviations

Any unforeseen and unavoidable deviations from the protocol will be documented and filed in a protocol deviation folder, with explanation.

Direct access to Source Data/Documents

Only the clinical team will have access to the participants' health records and personal information. However, the PI will provide direct access to the source data documents to the ethics committee should this be requested, to the regulatory agency, and to authorised representatives of the sponsor in order to permit trial related monitoring and audits.

13. STATISTICS

Sample Size Considerations

The efficacious malaria vaccine RTS,S/AS02 has been subjected to sporozoite challenge studies in non-immune volunteers (62) as well as field trials in adults in the Gambia (30) In sporozoite challenge, RTS,S/AS02 appears to give 32% sterile protection and 30% partial protection. Efficacy was higher in semi-immune adults in field studies, with 70% during the first 9 weeks of follow up.

Vaccine efficacy of ChAd63/MVA ME-TRAP in sporozoite challenge studies in Oxford was 21% sterile protection with 36% partial protection. We therefore consider it plausible that ChAd63/MVA ME-TRAP might show 50% efficacy in field studies of adults.

Assuming 50% efficacy of vaccination, with an estimated 70% local incidence of malaria by PCR during the follow up period, we expect 93% power to detect, at p=0.05, an efficacy of 50% and 77% power to detect an efficacy of 40% with 100 volunteers. Allowing for up to 20 volunteers to be lost to follow up, our sample size would be 120.

Below we tabulate the power obtained with differing vaccine efficacies and rates of PCR positivity, assuming p=0.05 and 100 evaluable volunteers completing the study.

It should be noted that the incidence of asymptomatic parasite carriage detected by qPCR was taken to be 70% on the basis of two observations in the literature.

a) The qPCR technique was used in the Gambia to assess the impact of prospective vaccines on 102 volunteers. The results of follow-up over five months showed that 71% of the volunteers became positive again at some time or other during follow-up (Imoukhuede et al. 2007[1]; see paper attached). We also include a second article by Andrews et al. highlighting the excellent sensitivity of this real-time PCR method[2].
b) The second point comes from a meta-analysis of a KEMRI study in Kenya. National Malaria Control Programme data put the estimated prevalence of asymptomatic parasite carriage detected by a rapid diagnostic test at between 26% and 32%. According to Okell et al.[3], there is a linear correlation between the percentage of positive adults in RDTs and the percentage of positive individuals in qualitative real-time PCR. In Kenya, a 30% positive RDT rate corresponded to a 60% positive PCR rate in a cross-sectional study. It is highly likely, therefore, that many more individuals would be positive if, instead of a study over time T, we proposed monitoring volunteers for six months, as the 70% positive rate found by Imoukhuede et al. confirms.

Table 8: *Power of study with n=100 tabulated by vaccine efficacy and prevalence of PCR positivity*

Prevalence of PCR positivity					
		0.8	0.7	0.6	0.5
Vaccine efficacy	50%	98	93	83	67
	40%	89	77	62	46
	30%	67	50	38	27

Planned Interim Analysis

[1] Imoukhuede EB, Andrews L, Milligan P, Berthoud T, Bojang K, Nwakanma D, Ismaili J, Buckee C, Njie F, Keita S, Sowe M, Lang T, Gilbert SC, Greenwood BM, Hill AV. 2007. Low-level malaria infections detected by a sensitive polymerase chain reaction assay and use of this technique in the evaluation of malaria vaccines in an endemic area. Am J Trop Med Hyg 76: 486-93.

[2] Andrews L, Andersen R, Webster D, et al. 2005. Quantitative real-time polymerase chain reaction for malaria diagnosis and its use in malaria vaccine clinical trials. Am J. Trop. Hyg. (73)1. pp. 191-198.

[3] Okell LC, Ghani AC, Lyons E, Drakeley CJ. 2009. Submicroscopic infection in *Plasmodium falciparum*-endemic populations: a systematic review and meta-analysis. J Infect Dis 200: 1509-17.

Safety Review
No interim safety analysis is planned.

Immunogenicity or Efficacy Review
No interim immunogenicity analysis is planned.

Final Analysis Plan
Analyses will be performed on subjects with intent to treat (ITT) and according to protocol (ATP).
Intent-to-treat cohort (ITT)

The intent-to-treat cohort will include all subjects enrolled in the study, who received at least one dose of the candidate malaria vaccines or comparator and for whom data for the observation in question are available.

Protocol-defined cohort for analysis of efficacy (ATP)

The protocol-defined cohort for analysis of efficacy will include all evaluable subjects (i.e., those meeting all eligibility criteria, complying with the procedures defined in the protocol, for whom data concerning efficacy endpoint measures are available.

Safety data

All reactogenicity analyses will be on an intention to treat basis, comparing control to malaria vaccine regimen. The frequency of adverse events, changes in ALT, plasma creatinine, haemoglobin, white cell count, platelet values will be described by time and vaccine group.

Immunogenicity

Both ITT and ATP cohorts will be analysed, using immediate *ex vivo* and cultured ELISpots, and ICS data. Geometric means and medians of T cell numbers with 95% confidence intervals for central tendency will describe groups.

Demographic data.

Age, location of residence, PCR at day 0 and bed net use will be presented by treatment group to ensure comparability.

Efficacy

Efficacy will be analysed both ITT and ATP. Cox regression model analysis of incidence of PCR positivity will allow assessment of the time to first episode as the primary outcome. Various thresholds for PCR positivity will be included for comparison. Age, bed net use and location of residence will be used as covariates. Immunological responses will also be examined as covariates to look for correlates of immunity. These analyses will also be undertaken as a metaanalysis of the combined results of VAC046 and VAC047.

The full analysis plan will be approved by the DSMB before implementation.

14. TIME FRAME

Pilot Study

Not applicable

Definitive study

Community sensitization, setting up of laboratory, test ELISPOTS, ICS and PCR assays, consent and screening procedures	April 2012 to July 2012
Vaccination and follow-up	Commencing July 2012
Data analysis	1st to 2nd Quarter 2013

15. ETHICAL CONSIDERATIONS

Human subjects

Good Clinical Practice

This trial will be conducted in accordance with the Declaration of Helsinki as agreed by the World Medical Association General Assembly (Seoul 2008), ICH Good Clinical Practice and local regulatory requirements.

Ethical Review

Before the inclusion of the first subject in the study, the protocol and the informed consent must be approved by the Ethical Review Committee in Senegal and the Oxford Tropical Research Ethics Committee (OXTREC).

The volunteer should give written informed consent before being included in the trial, after having been informed of the nature of the trial, the potential risks and their obligations. Informed consent forms will be provided in duplicate (original kept by the investigator, one copy kept by the subject or the subject's legally acceptable representative).

Confidentiality

Personal information of the volunteers will be handled confidentially. The principal Investigator will ensure confidentiality of personal data in accordance with ICH GCP and the Declaration of Helsinki (including staff handling the data). Blood samples will be coded with the volunteers study identification number and no personal details will be included so that information can not

be linked to the volunteers. Blood test results will be linked to personal identifying information only on documents held securely in the Clinical Trials Facility. No personal information will be entered into the electronic database, which will identify subjects by their unique code number only. All HIV tests and HIV related referrals will be handled with particular sensitivity. They will not be recorded in the CRF, but will be held securely in a separate document held by the Principal Investigator.
Any future research related to the data or samples from this study will require written scientific and ERC approval before it can be done.

Feedback of information

After the study we expect to hold a series of meetings with initial groups involved with sensitization, to relay feedback on the key findings of this study.

What risks may be involved?

The general risks to participants are those associated with blood sampling and vaccination. Mild local tenderness and bruising, light-headedness, or rarely vasovagal syncope, may result from venepuncture. As with any invasive procedure, infection is also a risk. This risk is minimized by the use of pre-packaged sterile equipment and careful attention to proper technique. There is no risk of HIV infection from phlebotomy since all needles are disposed of after a single use. While performing the procedure, loss of vacuum or collapsed vein might necessitate another draw.

Potential risks from vaccination include local and systemic reactions, which shall be solicited for in this study. Local symptoms including discomfort/pain, redness, swelling, and tenderness at the site of injection may be experienced by the volunteers. Systemic symptoms including myalgia, arthralgia, fatigue, malaise, headache, nausea, vomiting, fever or feverishness may be experienced by the volunteers.

ChAd63 ME-TRAP

To date, 186 volunteers have received ChAd63 ME-TRAP. Local adverse events such as pain would be expected to occur frequently. Less frequent local adverse events are likely to include erythema, swelling and warmth. Local adverse events are likely to be mild in nature and should resolve rapidly. Common systemic adverse events post viral vectored vaccines include headache, feverishness, myalgia, arthralgia, fatigue and malaise. Generally volunteers report a transient flu like illness within 24 hours of vaccination with ChAd63 which resolves completely within 48hrs. It is anticipated that the majority of systemic adverse events post ChAd63 ME-TRAP will be mild in intensity. No vaccine related serious adverse events have been seen with ChAd63 ME-TRAP.

During the manufacturing process of ChAd63 CS, a biocide named Kathon is used. Kathon is added to body washes, conditioners, liquid soaps, shampoos and wipes as a preservative. The

maximum dose is 0.1% for ‘rinse off’ products and for ‘leave on’ products it is 0.05%. It has been approved by regulatory authorities throughout the world as a preservative in these products. As a skin sensitiser it is known to cause contact dermatitis. An internal study was set up to look at the levels of Kathon that were removed during the final purification step of buffer exchange. This study utilized high performance liquid chromatography and showed that trace amounts of Kathon may be left on the column after carrying out the rinse and sanitisation steps. However, the study confirmed greater than 99.9975% removal of Kathon to approximately 30 fold less than the limits for ‘leave on’ products containing Kathon. We will exclude anyone from the study with a history of clinically significant contact dermatitis or sensitivity to Kathon.

There are no additional safety concerns arising from clinical trial experience with the administration repeated doses of ChAd63 ME-TRAP. As described in the Investigator Brochure for ChAd63 ME-TRAP, five volunteers have received a second dose of ChAd63 ME-TRAP after vaccination with ChAd63 ME-TRAP followed by MVA ME-TRAP (VAC033 clinical trial, completed). In the ongoing VAC043 clinical trial, 12 volunteers have received two sequential doses of ChAd63 ME-TRAP and a further 6 have received three sequential doses.

MVA ME-TRAP

More than 700 volunteers have received MVA ME-TRAP in the United Kingdom (UK), Senegal and the Gambia. Most volunteers developed erythema (or discolouration in African skin) and swelling post vaccination which in the majority of cases was mild to moderate in nature. This was accompanied by discomfort, warmth, itching and scaling which typically resolved to a faint flat papule. Intramuscular administration of MVA ME-TRAP has significantly less local reactogenicity than intradermal. Local AEs are likely to be mild in nature and should resolve rapidly, although there is the possibility of moderate arm pain in some cases. Common systemic adverse events post MVA vectored vaccines include headache, feverishness, myalgia, arthralgia, fatigue, and malaise. Generally volunteers report a transient flu like illness within 24 hours of vaccination with MVA which resolves completely. It is anticipated that the majority of systemic adverse events post MVA ME-TRAP will be mild or moderate in intensity. No vaccine related serious adverse events have been seen with MVA ME-TRAP.

Rabies vaccine

Mild and self-limited local reactions such as pain at the site of injection, redness and swelling occur in 21–74% of vaccinees. Bleomycin and neomycin are used in the manufacturing process and therefore volunteers with a history of hypersensitivity to these drugs, or aminoglycoside antibiotics, will be excluded from the trial. Mild systemic reactions such as fever, headache, dizziness and gastrointestinal symptoms occur in 5–40%, and systemic hypersensitivity following booster injections in 6% of vaccinees, but are less common following primary immunization.

Duty to minimise risks to volunteers

Adverse events are possible with any new vaccine. The risk is minimised by use of two candidate vectors chosen specifically for their good safety profile. Nevertheless, this study is designed to employ small numbers of closely supervised vaccinations. The comprehensive quality control, toxicology and manufacturing data and the absence of vaccine related serious adverse events with ChAd63-MVA ME-TRAP in phase I studies in Oxford, Kenya and the Gambia provide evidence of the minimal nature of risk in this study. However anaphylaxis is estimated to occur at a frequency at 1 in 10^5 to 10^6 with all vaccines. Should such a rare event occur, resuscitation facilities will be available to manage the reaction. As with any vaccine, Guillain-Barré syndrome or immune-mediated reactions that can lead to organ damage including serious allergic reactions may occur but this should be extremely rare has not been seen to date with ChAd63-MVA ME-TRAP.

Benefits of participating in this study

There are no direct benefits to individuals participating in this phase II trial other than information about the volunteers' health status, and free medical care during the trial for acute illness.

In terms of protection against malaria, this vaccination approach has shown some evidence of protecting malaria naïve volunteers and therefore, may be protective against naturally occurring malaria infection in Senegal. However, because malaria mainly affects young children, the benefits of participating in this study may be considered as being altruistic in nature as they would potentially benefit the wider society at large, if the vaccine is eventually proved to be safe and efficacious.

Those receiving the comparator vaccine, Rabies vaccine, will benefit from some protection against rabies. All participants will be offered a full standard schedule of Rabies vaccine at the end of the trial in order to guarantee vaccine efficacy and to avoid inequality amongst participants. (These vaccinations will not be considered study procedures).

There will be a perception of benefit from physical examination and laboratory screening. We will also offer compensation for transport expenses, and time taken away from work in order to attend the scheduled clinic visits. We believe that compensation for the loss of time, transport expenses and inconvenience of study visits is required. This will be paid per clinic visit, rather than as lump sums, to avoid coercion to complete the study. Based on local wages and in line with other studies in the region, we suggest that 5,000 CFA francs be paid per study visit. The payment amount will be approved by the Ethics Committee prior to the commencement of the study.

The amount takes into consideration any lost wages for attending to the study while ensuring that it neither unduly coerces potential participants nor sets a difficult precedent for all research done within the programme. We believe that these benefits will not be excessive, and believe it would be unreasonable to request the cooperation of a population in regular employment without jeopardising their regular productivity or income.

Duty to assure high standards of informed consent

We will provide detailed information about the study for distribution to volunteers. The principal investigator will endeavour to ensure that all volunteers fully understand the risks. Any participant who appears to have less than complete understanding will be excluded from enrolment by the principal investigator. As with any experimental vaccine the participants must understand that they have not yet been shown to prevent infection and this will be stressed during the recruitment stage. They must also understand the very small chance of anaphylactic reactions and thereby the importance of complying with the 60 minute observation period after each vaccination. The information sheet (attached) covers these points in detail, and each participant will have attended a public meeting, had the contents of the sheet explained in individual meetings on 2 separate occasions and comprehension assessed prior to signing the consent form.

Animal subjects

Not applicable

16. EXPECTED APPLICATION OF RESULTS

Data from this study will provide further information about the efficacy, safety and immunogenicity of these new promising vaccines, and such studies are essential for developing effective malaria vaccines. Findings would potentially benefit the wider community in allowing vaccine programmes for larger populations to progress. Intellectual Property Rights (IPR) issues will be dealt with according to the Ucad IPR policy.

17. REFERENCES

1. Okiro EA, Hay SI, Gikandi PW, Sharif SK, Noor AM, Peshu N, Marsh K, Snow RW. 2007. The decline in paediatric malaria admissions on the coast of Kenya. *Malar J* 6: 151
2. Sievers AC, Lewey J, Musafiri P, Franke MF, Bucyibaruta BJ, Stulac SN, Rich ML, Karema C, Daily JP. 2008. Reduced paediatric hospitalizations for malaria and febrile illness patterns following implementation of community-based malaria control programme in rural Rwanda. *Malar J* 7: 167
3. O'Meara WP, Mwangi TW, Williams TN, McKenzie FE, Snow RW, Marsh K. 2008. Relationship between exposure, clinical malaria, and age in an area of changing transmission intensity. *Am J Trop Med Hyg* 79: 185-91

4. Ceesay SJ, Casals-Pascual C, Erskine J, Anya SE, Duah NO, Fulford AJ, Sesay SS, Abubakar I, Dunyo S, Sey O, Palmer A, Fofana M, Corrah T, Bojang KA, Whittle HC, Greenwood BM, Conway DJ. 2008. Changes in malaria indices between 1999 and 2007 in The Gambia: a retrospective analysis. *Lancet* 372: 1545-54
5. O'Meara WP, Bejon P, Mwangi TW, Okiro EA, Peshu N, Snow RW, Newton CR, Marsh K. 2008. Effect of a fall in malaria transmission on morbidity and mortality in Kilifi, Kenya. *Lancet* 372: 1555-62
6. Okiro EA, Alegana VA, Noor AM, Snow RW. 2010. Changing malaria intervention coverage, transmission and hospitalization in Kenya. *Malar J* 9: 285
7. Steketee RW, Campbell CC. 2010. Impact of national malaria control scale-up programmes in Africa: magnitude and attribution of effects. *Malar J* 9: 299
8. Greenwood BM, Fidock DA, Kyle DE, Kappe SH, Alonso PL, Collins FH, Duffy PE. 2008. Malaria: progress, perils, and prospects for eradication. *J Clin Invest* 118: 1266-76
9. Snow RW, Guerra CA, Noor AM, Myint HY, Hay SI. 2005. The global distribution of clinical episodes of Plasmodium falciparum malaria. *Nature* 434: 214-7
10. Snow RW, Craig M, Deichmann U, Marsh K. 1999. Estimating mortality, morbidity and disability due to malaria among Africa's non-pregnant population. *Bull World Health Organ* 77: 624-40
11. Stapleton DH. 2004. Lessons of history? Anti-malaria strategies of the International Health Board and the Rockefeller Foundation from the 1920s to the era of DDT. *Public Health Rep* 119: 206-15
12. Carter ED. 2009. "God bless General Peron": DDT and the endgame of malaria eradication in Argentina in the 1940s. *J Hist Med Allied Sci* 64: 78-122
13. Bruce-Chwatt LJ. 1987. Malaria and its control: present situation and future prospects. *Annu Rev Public Health* 8: 75-110
14. Feachem RG, Phillips AA, Targett G, Group TME. 2009. *Shrinking the malaria map*. San Fransisco: The Global Health Group
15. Penny MA, Maire N, Studer A, Schapira A, Smith TA. 2008. What should vaccine developers ask? Simulation of the effectiveness of malaria vaccines. *PLoS One* 3: e3193
16. Bejon P, Lusingu J, Olotu A, Leach A, Lievens M, Vekemans J, Mshamu S, Lang T, Gould J, Dubois MC, Demoitie MA, Stallaert JF, Vansadia P, Carter T, Njuguna P, Awuondo KO, Malabeja A, Abdul O, Gesase S, Mturi N, Drakeley CJ, Savarese B, Villafana T, Ballou WR, Cohen J, Riley EM, Lemnge MM, Marsh K, von Seidlein L. 2008. Efficacy of RTS,S/AS01E vaccine against malaria in children 5 to 17 months of age. *N Engl J Med* 359: 2521-32
17. Olotu A, Lusingu J, Leach A, Lievens M, Vekemans J, Msham S, Lang T, Gould J, Dubois MC, Jongert E, Vansadia P, Carter T, Njuguna P, Awuondo KO, Malabeja A, Abdul O, Gesase S, Mturi N, Drakeley CJ, Savarese B, Villafana T, Lapierre D, Ballou WR, Cohen J, Lemnge MM, Peshu N, Marsh K, Riley EM, von Seidlein L, Bejon P. 2011. Efficacy of RTS,S/AS01E malaria vaccine and exploratory analysis on anti-circumsporozoite antibody titres and protection in children aged 5-17 months in Kenya and Tanzania: a randomised controlled trial. *Lancet Infect Dis* 11: 102-9
18. Moorthy VS, Pinder M, Reece WH, Watkins K, Atabani S, Hannan C, Bojang K, McAdam KP, Schneider J, Gilbert S, Hill AV. 2003. Safety and immunogenicity of DNA/modified vaccinia virus ankara malaria vaccination in African adults. *J Infect Dis* 188: 1239-44
19. Webster DP, Dunachie S, Vuola JM, Berthoud T, Keating S, Laidlaw SM, McConkey SJ, Poulton I, Andrews L, Andersen RF, Bejon P, Butcher G, Sinden R, Skinner MA, Gilbert SC, Hill AV. 2005. Enhanced T cell-mediated protection against malaria in human challenges by using the recombinant poxviruses FP9 and modified vaccinia virus Ankara. *Proc Natl Acad Sci U S A* 102: 4836-41

20. Bejon P, Kai OK, Mwacharo J, Keating S, Lang T, Gilbert SC, Peshu N, Marsh K, Hill AV. 2006. Alternating vector immunizations encoding pre-erythrocytic malaria antigens enhance memory responses in a malaria endemic area. *Eur J Immunol* 36: 2264-72
21. Reyes-Sandoval A, Sridhar S, Berthoud T, Moore AC, Harty JT, Gilbert SC, Gao G, Ertl HC, Wilson JC, Hill AV. 2008. Single-dose immunogenicity and protective efficacy of simian adenoviral vectors against Plasmodium berghei. *Eur J Immunol* 38: 732-41
22. Sridhar S, Reyes-Sandoval A, Draper SJ, Moore AC, Gilbert SC, Gao GP, Wilson JM, Hill AV. 2008. Single-dose protection against Plasmodium berghei by a simian adenovirus vector using a human cytomegalovirus promoter containing intron A. *J Virol* 82: 3822-33
23. Bejon P, Peshu N, Gilbert SC, Lowe BS, Molyneux CS, Forsdyke J, Lang T, Hill AV, Marsh K. 2006. Safety profile of the viral vectors of attenuated fowlpox strain FP9 and modified vaccinia virus Ankara recombinant for either of 2 preerythrocytic malaria antigens, ME-TRAP or the circumsporozoite protein, in children and adults in Kenya. *Clin Infect Dis* 42: 1102-10
24. Bejon P, Mwacharo J, Kai O, Mwangi T, Milligan P, Todryk S, Keating S, Lang T, Lowe B, Gikonyo C, Molyneux C, Fegan G, Gilbert SC, Peshu N, Marsh K, Hill AV. 2006. A phase 2b randomised trial of the candidate malaria vaccines FP9 ME-TRAP and MVA ME-TRAP among children in Kenya. *PLoS Clin Trials* 1: e29
25. Moorthy VS, Imoukhuede EB, Milligan P, Bojang K, Keating S, Kaye P, Pinder M, Gilbert SC, Walraven G, Greenwood BM, Hill AS. 2004. A randomised, double-blind, controlled vaccine efficacy trial of DNA/MVA ME-TRAP against malaria infection in Gambian adults. *PLoS Med* 1: e33
26. Osman MM, Nour BY, Sedig MF, De Bes L, Babikir AM, Mohamedani AA, Mens PF. 2010. Informed decision-making before changing to RDT: a comparison of microscopy, rapid diagnostic test and molecular techniques for the diagnosis and identification of malaria parasites in Kassala, eastern Sudan. *Trop Med Int Health* 15: 1442-8
27. Rantala AM, Taylor SM, Trottman PA, Luntamo M, Mbewe B, Maleta K, Kulmala T, Ashorn P, Meshnick SR. 2010. Comparison of real-time PCR and microscopy for malaria parasite detection in Malawian pregnant women. *Malar J* 9: 269
28. Bejon P, Andrews L, Andersen RF, Dunachie S, Webster D, Walther M, Gilbert SC, Peto T, Hill AV. 2005. Calculation of liver-to-blood inocula, parasite growth rates, and preerythrocytic vaccine efficacy, from serial quantitative polymerase chain reaction studies of volunteers challenged with malaria sporozoites. *J Infect Dis* 191: 619-26
29. Imoukhuede EB, Andrews L, Milligan P, Berthoud T, Bojang K, Nwakanma D, Ismaili J, Buckee C, Njie F, Keita S, Sowe M, Lang T, Gilbert SC, Greenwood BM, Hill AV. 2007. Low-level malaria infections detected by a sensitive polymerase chain reaction assay and use of this technique in the evaluation of malaria vaccines in an endemic area. *Am J Trop Med Hyg* 76: 486-93
30. Bojang KA, Milligan PJ, Pinder M, Vigneron L, Alloueche A, Kester KE, Ballou WR, Conway DJ, Reece WH, Gothard P, Yamuah L, Delchambre M, Voss G, Greenwood BM, Hill A, McAdam KP, Tornieporth N, Cohen JD, Doherty T. 2001. Efficacy of RTS,S/AS02 malaria vaccine against Plasmodium falciparum infection in semi-immune adult men in The Gambia: a randomised trial. *Lancet* 358: 1927-34
31. Mayr A, Stickl H, Muller HK, Danner K, Singer H. 1978. [The smallpox vaccination strain MVA: marker, genetic structure, experience gained with the parenteral vaccination and behavior in organisms with a debilitated defence mechanism (author's transl)]. *Zentralbl Bakteriol B* 167: 375-90
32. Mayr A, Stickl H, Muller HK, Danner K, Singer H. 1978. [The smallpox vaccination strain MVA: marker, genetic structure, experience gained with the parenteral vaccination and behavior in

organisms with a debilitated defence mechanism (author's transl)]. *Zentralbl Bakteriol [B]* 167: 375-90
33. Mahnel H, Mayr A. 1994. [Experiences with immunization against orthopox viruses of humans and animals using vaccine strain MVA]. *Berl Munch Tierarztl Wochenschr* 107: 253-6
34. Stickl H, Hochstein-Mintzel V, Mayr A, Huber HC, Schafer H, Holzner A. 1974. [MVA vaccination against smallpox: clinical tests with an attenuated live vaccinia virus strain (MVA) (author's transl)]. *Dtsch Med Wochenschr* 99: 2386-92
35. Hanke T, McMichael AJ, Dennis MJ, Sharpe SA, Powell LA, McLoughlin L, Crome SJ. 2005. Biodistribution and persistence of an MVA-vectored candidate HIV vaccine in SIV-infected rhesus macaques and SCID mice. *Vaccine* 23: 1507-14
36. Howles S, Guimaraes-Walker A, Yang H, Hancock G, di Gleria K, Tarragona-Fiol T, Hayes P, Gilmour J, Bridgeman A, Hanke T, McMichael A, Dorrell L. 2010. Vaccination with a modified vaccinia virus Ankara (MVA)-vectored HIV-1 immunogen induces modest vector-specific T cell responses in human subjects. *Vaccine* 28: 7306-12
37. Goepfert PA, Elizaga ML, Sato A, Qin L, Cardinali M, Hay CM, Hural J, DeRosa SC, DeFawe OD, Tomaras GD, Montefiori DC, Xu Y, Lai L, Kalams SA, Baden LR, Frey SE, Blattner WA, Wyatt LS, Moss B, Robinson HL. 2011. Phase 1 safety and immunogenicity testing of DNA and recombinant modified vaccinia Ankara vaccines expressing HIV-1 virus-like particles. *J Infect Dis* 203: 610-9
38. Hawkridge T, Scriba TJ, Gelderbloem S, Smit E, Tameris M, Moyo S, Lang T, Veldsman A, Hatherill M, Merwe L, Fletcher HA, Mahomed H, Hill AV, Hanekom WA, Hussey GD, McShane H. 2008. Safety and immunogenicity of a new tuberculosis vaccine, MVA85A, in healthy adults in South Africa. *J Infect Dis* 198: 544-52
39. Berthoud TK, Hamill M, Lillie PJ, Hwenda L, Collins KA, Ewer KJ, Milicic A, Poyntz HC, Lambe T, Fletcher HA, Hill AV, Gilbert SC. 2011. Potent CD8+ T-cell immunogenicity in humans of a novel heterosubtypic influenza A vaccine, MVA-NP+M1. *Clin Infect Dis* 52: 1-7
40. Dangoor A, Lorigan P, Keilholz U, Schadendorf D, Harris A, Ottensmeier C, Smyth J, Hoffmann K, Anderson R, Cripps M, Schneider J, Hawkins R. 2010. Clinical and immunological responses in metastatic melanoma patients vaccinated with a high-dose poly-epitope vaccine. *Cancer Immunol Immunother* 59: 863-73
41. Dunachie SJ, Walther M, Vuola JM, Webster DP, Keating SM, Berthoud T, Andrews L, Bejon P, Poulton I, Butcher G, Watkins K, Sinden RE, Leach A, Moris P, Tornieporth N, Schneider J, Dubovsky F, Tierney E, Williams J, Heppner DG, Jr., Gilbert SC, Cohen J, Hill AV. 2006. A clinical trial of prime-boost immunisation with the candidate malaria vaccines RTS,S/AS02A and MVA-CS. *Vaccine* 24: 2850-9
42. Dunachie SJ, Walther M, Epstein JE, Keating S, Berthoud T, Andrews L, Andersen RF, Bejon P, Goonetilleke N, Poulton I, Webster DP, Butcher G, Watkins K, Sinden RE, Levine GL, Richie TL, Schneider J, Kaslow D, Gilbert SC, Carucci DJ, Hill AV. 2006. A DNA prime-modified vaccinia virus ankara boost vaccine encoding thrombospondin-related adhesion protein but not circumsporozoite protein partially protects healthy malaria-naive adults against Plasmodium falciparum sporozoite challenge. *Infect Immun* 74: 5933-42
43. Top FH, Jr., Buescher EL, Bancroft WH, Russell PK. 1971. Immunization with live types 7 and 4 adenovirus vaccines. II. Antibody response and protective effect against acute respiratory disease due to adenovirus type 7. *J Infect Dis* 124: 155-60
44. Tatsis N, Ertl HC. 2004. Adenoviruses as vaccine vectors. *Mol Ther* 10: 616-29
45. Kobinger GP, Feldmann H, Zhi Y, Schumer G, Gao G, Feldmann F, Jones S, Wilson JM. 2006. Chimpanzee adenovirus vaccine protects against Zaire Ebola virus. *Virology* 346: 394-401
46. Casimiro DR, Chen L, Fu TM, Evans RK, Caulfield MJ, Davies ME, Tang A, Chen M, Huang L, Harris V, Freed DC, Wilson KA, Dubey S, Zhu DM, Nawrocki D, Mach H, Troutman R, Isopi L, Williams D,

Hurni W, Xu Z, Smith JG, Wang S, Liu X, Guan L, Long R, Trigona W, Heidecker GJ, Perry HC, Persaud N, Toner TJ, Su Q, Liang X, Youil R, Chastain M, Bett AJ, Volkin DB, Emini EA, Shiver JW. 2003. Comparative immunogenicity in rhesus monkeys of DNA plasmid, recombinant vaccinia virus, and replication-defective adenovirus vectors expressing a human immunodeficiency virus type 1 gag gene. *J Virol* 77: 6305-13

47. Xiang Z, Gao G, Reyes-Sandoval A, Cohen CJ, Li Y, Bergelson JM, Wilson JM, Ertl HC. 2002. Novel, chimpanzee serotype 68-based adenoviral vaccine carrier for induction of antibodies to a transgene product. *J Virol* 76: 2667-75
48. Catanzaro AT, Koup RA, Roederer M, Bailer RT, Enama ME, Moodie Z, Gu L, Martin JE, Novik L, Chakrabarti BK, Butman BT, Gall JG, King CR, Andrews CA, Sheets R, Gomez PL, Mascola JR, Nabel GJ, Graham BS. 2006. Phase 1 safety and immunogenicity evaluation of a multiclade HIV-1 candidate vaccine delivered by a replication-defective recombinant adenovirus vector. *J Infect Dis* 194: 1638-49
49. Harro CD, Robertson MN, Lally MA, O'Neill LD, Edupuganti S, Goepfert PA, Mulligan MJ, Priddy FH, Dubey SA, Kierstead LS, Sun X, Casimiro DR, DiNubile MJ, Shiver JW, Leavitt RY, Mehrotra DV. 2009. Safety and immunogenicity of adenovirus-vectored near-consensus HIV type 1 clade B gag vaccines in healthy adults. *AIDS Res Hum Retroviruses* 25: 103-14
50. Tatsis N, Blejer A, Lasaro MO, Hensley SE, Cun A, Tesema L, Li Y, Gao GP, Xiang ZQ, Zhou D, Wilson JM, Ertl HC. 2007. A CD46-binding chimpanzee adenovirus vector as a vaccine carrier. *Mol Ther* 15: 608-17
51. Dudareva M, Andrews L, Gilbert SC, Bejon P, Marsh K, Mwacharo J, Kai O, Nicosia A, Hill AV. 2009. Prevalence of serum neutralizing antibodies against chimpanzee adenovirus 63 and human adenovirus 5 in Kenyan children, in the context of vaccine vector efficacy. *Vaccine* 27: 3501-4
52. Gilbert SC, Plebanski M, Harris SJ, Allsopp CE, Thomas R, Layton GT, Hill AV. 1997. A protein particle vaccine containing multiple malaria epitopes. *Nat Biotechnol* 15: 1280-4
53. Robson KJ, Hall JR, Jennings MW, Harris TJ, Marsh K, Newbold CI, Tate VE, Weatherall DJ. 1988. A highly conserved amino-acid sequence in thrombospondin, properdin and in proteins from sporozoites and blood stages of a human malaria parasite. *Nature* 335: 79-82
54. Lalvani A, Aidoo M, Allsopp CE, Plebanski M, Whittle HC, Hill AV. 1994. An HLA-based approach to the design of a CTL-inducing vaccine against Plasmodium falciparum. *Res Immunol* 145: 461-8
55. Rogers WO, Malik A, Mellouk S, Nakamura K, Rogers MD, Szarfman A, Gordon DM, Nussler AK, Aikawa M, Hoffman SL. 1992. Characterization of Plasmodium falciparum sporozoite surface protein 2. *Proc Natl Acad Sci U S A* 89: 9176-80
56. Walther M, Thompson FM, Dunachie S, Keating S, Todryk S, Berthoud T, Andrews L, Andersen RF, Moore A, Gilbert SC, Poulton I, Dubovsky F, Tierney E, Correa S, Huntcooke A, Butcher G, Williams J, Sinden RE, Hill AV. 2006. Safety, immunogenicity, and efficacy of prime-boost immunization with recombinant poxvirus FP9 and modified vaccinia virus Ankara encoding the full-length Plasmodium falciparum circumsporozoite protein. *Infect Immun* 74: 2706-16
57. Plebanski M, Gilbert SC, Schneider J, Hannan CM, Layton G, Blanchard T, Becker M, Smith G, Butcher G, Sinden RE, Hill AV. 1998. Protection from Plasmodium berghei infection by priming and boosting T cells to a single class I-restricted epitope with recombinant carriers suitable for human use. *Eur J Immunol* 28: 4345-55
58. Reece WH, Pinder M, Gothard PK, Milligan P, Bojang K, Doherty T, Plebanski M, Akinwunmi P, Everaere S, Watkins KR, Voss G, Tornieporth N, Allloueche A, Greenwood BM, Kester KE, McAdam KP, Cohen J, Hill AV. 2004. A CD4(+) T-cell immune response to a conserved epitope in the circumsporozoite protein correlates with protection from natural Plasmodium falciparum infection and disease. *Nat Med* 10: 406-10

59. Keating SM, Bejon P, Berthoud T, Vuola JM, Todryk S, Webster DP, Dunachie SJ, Moorthy VS, McConkey SJ, Gilbert SC, Hill AV. 2005. Durable human memory T cells quantifiable by cultured enzyme-linked immunospot assays are induced by heterologous prime boost immunization and correlate with protection against malaria. *J Immunol* 175: 5675-80
60. Schofield L, Villaquiran J, Ferreira A, Schellekens H, Nussenzweig R, Nussenzweig V. 1987. Gamma interferon, CD8+ T cells and antibodies required for immunity to malaria sporozoites. *Nature* 330: 664-6
61. Okell LC, Ghani AC, Lyons E, Drakeley CJ. 2009. Submicroscopic infection in Plasmodium falciparum-endemic populations: a systematic review and meta-analysis. *J Infect Dis* 200: 1509-17
62. Kester KE, Cummings JF, Ofori-Anyinam O, Ockenhouse CF, Krzych U, Moris P, Schwenk R, Nielsen RA, Debebe Z, Pinelis E, Juompan L, Williams J, Dowler M, Stewart VA, Wirtz RA, Dubois MC, Lievens M, Cohen J, Ballou WR, Heppner DG, Jr. 2009. Randomized, double-blind, phase 2a trial of falciparum malaria vaccines RTS,S/AS01B and RTS,S/AS02A in malaria-naive adults: safety, efficacy, and immunologic associates of protection. *J Infect Dis* 200: 337-46
63. Plotkin SA, Wiktor T. Rabies Vaccination. *Ann Rev Med* 1978; 29:583-91.
64. Reyes-Sandoval A, Berthoud T, Alder N, Siani L, Gilbert SC, Nicosia A, Colloca S, Cortese R, Hill AVS. Prime-Boost Immunization with Adenoviral and Modified Vaccinia Virus Ankara Vectors Enhances the Durability and Polyfunctionality of Protective Malaria $CD8^{+}$ T-Cell Responses. Infection and Immunity 2010 Jan; 78(1): 145-153.
65. Guidelines for the Treatment of Malaria. Second Edition. World Health Organization. 2010.

18. FINANCE

Justification of the Budget

The resources requested are for the proper conduct of the phase II trials and related activities in Senegal. All salaries are based on UCAD, Senegal pay scales. Study clinicians are needed to conduct the trial under the supervision of the PIs. 1 nurse, 1 clinical officer, 1 medical officer, and 8 field workers are needed to help with the recruitment and follow-up. Three laboratory technicians are needed to provide laboratory support for the study. A full time driver is needed to help with transportation of staff and study subjects for the duration of the study. A project coordinator is needed to ensure that all procedures and their documentation meet GCP and good clinical laboratory practice (GCLP) quality standards. 1 data manager will be needed for data management and 2 data clerks for data entry.

This study is part of the UCAD research Programme and will not incur any consultancy fees or additional administrative overheads.

19. PROTOCOL VERSION HISTORY

The version history of this protocol, along with descriptions of changes made, is detailed below.

Protocol version, date	Description of change/s from previous version		
	Section	Subsection, page (of tracked version)	Change/s
1.0, 22 December 2011			not applicable
2.0, 18 March 2012	19	p 49	Added this section, "Protocol Version History"
	Title page	p1	Addition of investigators
	5	"Rationale", p10	Changed to reflect change in vaccination regimen and addition of metaanalysis with the parallel study, VAC046.
	9	"Endpoints", p29-30	Revised endpoints for the addition of metaanalysis with VAC046 as a study objective
	13	p39	Addition of metaanalysis to analysis of efficacy
	8	p12	Added metaanalysis with VAC046 as a study objective
	9	"Study Groups" p 16	Changed to state change in vaccination regimen
	9	"Study Groups' p16-17; "Follow-up", p19; "Blood Sampling" p 20-21; "Laboratory Procedures" p 23	Changed to state new timeline of study visits, consequent to change in vaccination regimen
		"Blood Sampling" p 21-23	Tables 2 and 3 changed to state new timeline of study visits and blood volumes, consequent to change in vaccination regimen
	9	"Blood Sampling" p 21)	Corrected typographical error: "10ml" rather than "5ml" of blood collected at Days 0, 14, 56 for Group B, as indicated on sampling volumes table. Corrected typographical error on Sampling volumes table: 10 ml taken from Group B volunteers at D161.
	9	Table 4 (p 24-26); "Study Visits" p 27-29	Changed to show new timeline of study visits and procedures, consequent to change in vaccination regimen.
	9	Study Site and population, p12-14;	Revised to describe new study site

		Methodology, p 15	
	9	"Sensitization and Recruitment" p16; "Study Schedule and Procedures" p19; Laboratory Procedures, p 23.	Change of stated study site
	13	"Sample size considerations", p37-38	Revised power calculations
	9	"Sensitisation and recruitment", p 15	Clarified that participants eligible to participate in the study are those that meet all of the inclusion criteria and none of the exclusion criteria.
	9	"Study Schedule and Procedures" p 18-19	Removed the following: "Volunteers who cannot write will be able to thumb print the consent form, which will be counter-signed by an independent witness."
	9	"Vaccine administration", p 23-24	Changed subsection to "Vaccine Handling and Administration" and added further detail on the security measures for vaccine management.
	15	"Confidentiality", p 40	Clarified details of storage of data
	12	"Data handling and record keeping", p36-37	Clarified details of storage of data
	14	p 39	Changed dates for the conduct of the trial
	9	"Vaccine Administration" p 24	Clarified that Rabies vaccine will be sourced locally.
		Throughout document	" HDCRV", changed to "Rabies Vaccine"
	9	"Study Schedule and Procedures" p 19	Corrected typographical error ("ChAd63 ME-TRAP" instead of "ChAd63")
	15	"Duty to minimise risks to volunteers", p 42	Corrected typographical error ("Kenya" instead of "Senegal")
	9	"Sensitization and recruitment", p 15	Corrected typographical error: "enrolment" (receipt of first vaccination), rather than "recruitment", will take place within 90 days of the Screening visit.
	9	"Blood sampling", p20	Corrected omission, that Hepatitis B surface antigen will be tested on blood collected at screening
2.1, 16 July 2012	2	page 1	Addition of names and roles of all staff working on the study
	11	page 35	Addition of listing of members of the Data

			Safety Monitoring Board. Addition of names and contact details of the internal and external study monitors.
	13	“Sample size considerations”, page 37	Addition of further justification for the anticipated local incidence of malaria in the control group
2.2, 4 August 2012	2	page 1	Addition of names and roles of all staff working on the study
	11	page 35	Change of member of DSMB
