# Supplementary material for: Safety, Immunogenicity and Efficacy of Prime-Boost Vaccination with ChAd63 and MVA Encoding ME-TRAP against Plasmodium falciparum Infection in Adults in Senegal
Source: PLoS One. 2016 Dec 15;11(12):e0167951. doi: 10.1371/journal.pone.0167951 (PMC5158312; doi:10.1371/journal.pone.0167951)
Supplement: S3 Appendix — (PDF) [file pone.0167951.s007.pdf]

**VAC047**		117 volunteers	**D0**	**D63**	**D70**	**D72**	**D74**	**D77**	**D79**	**D81**	**D84**	**D86**	**D88**	**D91**	**D93**	**D95**	**D98**	**D105**	**D112**	**D119**
		volunteers PCR positive	8	11	4	5	7	2	5	5	4	6	5	6	7	7	4	4	4	3
No. volunteers with ANY PCR +ve	27	% of total	6.84	9.40	3.42	4.27	5.98	1.71	4.27	4.27	3.42	5.13	4.27	5.13	5.98	5.98	3.42	3.42	3.42	2.56
No. volunteers with ANY >D70 +ves	21																			
No. volunteers with >1 post D70 PCR +ve	15																			

**VAC046**		118 volunteers	**D0**	**D63**	**D70**	**D72**	**D74**	**D77**	**D79**	**D81**	**D84**	**D86**	**D88**	**D91**	**D93**	**D95**	**D98**	**D105**	**D112**	**D119**
		volunteers PCR positive	78	46	23	20	18	4	5	4	2	2	3	0	0	0	2	4	2	1
No. volunteers with ANY PCR +ve	90	% of total	66.10	38.98	19.49	16.95	15.25	3.39	4.24	3.39	1.69	1.69	2.54	0.00	0.00	0.00	1.69	3.39	1.69	0.85
No. volunteers with ANY >D70 +ves	29																			
No. volunteers with >1 post D70 PCR +ve	14																			

**VAC047**	**D0**	**D63**	**D70**
number PCR +ve	8	11	4
%	6.84	9.40	3.42

8 pos @ D63 -ve at D70 (plus 3 +ve @ both D63 and D70)
(took a bit longer to clear, most early scores low)

**VAC046**	**D0**	**D63**	**D70**
number PCR +ve	78	46	23
%	66.10	38.98	19.49

(many more +ve early on)
32 pos @ D63 and -ve @ D70, plus 15 +ve @ both D63 and D70
(possibly more took longer to clear with typically higher early PCR scores)
